# Interventions that strengthen the care workforce: a realist synthesis review

**DOI:** 10.1093/haschl/qxaf128

**Published:** 2025-06-28

**Authors:** Christine Kelly, Lisette Dansereau, Ellie Jack, Salina Pirzada, Yuns Oh, Pranav Bhushan, Lorine Pelly, Janice Linton, Carey McCarthy, Giorgio Cometto

**Affiliations:** Department of Community Health Sciences, University of Manitoba, Winnipeg, MB, R3E 0W3, Canada; Department of Community Health Sciences, University of Manitoba, Winnipeg, MB, R3E 0W3, Canada; Department of Community Health Sciences, University of Manitoba, Winnipeg, MB, R3E 0W3, Canada; Department of Community Health Sciences, University of Manitoba, Winnipeg, MB, R3E 0W3, Canada; Department of Community Health Sciences, University of Manitoba, Winnipeg, MB, R3E 0W3, Canada; Institute for Global Public Health, University of Manitoba, Winnipeg, MB, R3E 0T6, Canada; Institute for Global Public Health, University of Manitoba, Winnipeg, MB, R3E 0T6, Canada; Neil John Maclean Health Sciences Library, University of Manitoba, Winnipeg, MB, R3E 3P5, Canada; Health Workforce Department, World Health Organization, 20 Avenue Appia, CH-1211 Geneva 27, Switzerland; Health Workforce Department, World Health Organization, 20 Avenue Appia, CH-1211 Geneva 27, Switzerland

**Keywords:** care workers, workforce planning, health human resources

## Abstract

**Introduction:**

Health systems depend on care workers to provide “hands-on” direct care with eating, dressing, and other needs, as well as indirect care with household tasks, meals, and transport. Care workers are in high demand to support growing populations who need help in daily life, yet they often fall outside of health human resource planning. Recruiting, supporting, and retaining the care workforce are urgent priorities for health workforce planners.

**Methods:**

This realist synthesis review asks: Which interventions strengthen the care workforce? We systematically identified 7396 peer-reviewed sources and 481 gray literature sources, with 151 included in the review.

**Results:**

The sources document a variety of interventions that strengthen the care workforce, with an emphasis on pre-service and ongoing training for care workers. There were ambitious interventions that aimed to support the care workforce on multiple fronts.

**Conclusion:**

Policy makers and researchers are encouraged to implement complex interventions that cover multiple factors simultaneously. We recommend focusing on legislative structures, educational oversight, and material working conditions, such as scheduling and pay, as highly promising avenues for strengthening the care workforce across multiple contexts.

Key Points

- **Key takeaway 1:** Despite increased demand for hands-on care workers, health workforce planning tends to overlook the contributions and needs of the *care workforce*.
- **Key takeaway 2:** Workforce interventions related to education and/or improving working conditions are key levers for strengthening the care workforce.
- **Key takeaway 3:** The findings support developing complex and ambitious interventions that aim to strengthen the care workforce in multiple ways across the entire working life span.

## Introduction

Health systems around the world are grappling with health workforce shortages compounded by rising population health needs.^1,2^ There is mounting evidence on health workforce issues such as strategic planning,^3^ regulation,^4^ recruitment, education, retention, and safety.^5^ Other work considers specific occupations such as social workers,^6^ nurses,^7^ physicians,^8^ and community health workers.^9^ Yet these efforts tend to overlook the roles and contributions of the frontline *care* workforce.

Health systems depend on care workers for “hands-on” direct care with eating, dressing, and other needs, and for indirect care such as household tasks, meals, and transport. Care workers have an array of job titles, including personal support workers, health care aides, and direct care workers. Care workers are in high demand to support those needing daily help due to disability, aging, acute or chronic health issues, inaccessible environments, and/or socio-economic vulnerabilities. Care workers are commonly found in home settings, residential care, and/or supportive roles in hospitals. Care workers “bridge the gap between regulated medical professionals and patients,”^10(p4)^ yet frequently fall outside of health human resource planning efforts. Care work is predominantly carried out by women, tends to be un- or underregulated, and is associated with low pay, high turnover, and precarious working conditions.^11^ Recruiting, supporting, and retaining—that is “strengthening”—the care workforce is an urgent priority for health workforce planners and for achieving health system and gender equality agendas.

### Objectives and focus of review

This work will be used to inform policy guidance for the planning, management, and support of an integrated health and care workforce. A foundational step is to improve our knowledge of care workforce issues by answering: **which interventions strengthen the care workforce?**

The review focuses on care workers who “provide direct personal care services in the home, in health care and residential settings, assisting with routine tasks of daily life, and performing a variety of other tasks of a simple and routine nature.”^12^ Most care work is unpaid (eg, tending to healthy children or unwell friends and family^11^); however, this review focuses on paid workers supporting those with care needs due to disability, aging, or health issues. See Appendix A1 for a detailed definition (to access the Appendix, click on the Details tab of the article online).

## Data and Methods

The review reports items from the RAMESES I and the PRISMA-S publication standards.^13-15^ Realist synthesis reviews examine evidence related to “complex interventions”.^16^ In this review, an intervention is understood to be any activity, policy, or legislation intended to produce an effect. Realist reviews explain which interventions work, for whom, and in which circumstances by identifying the context-mechanism-outcome (CMO) configuration.^13,14^ The CMO configuration is as follows:

Context: The country, scope, and service setting of the intervention.

Mechanism: The inferred or explicitly stated theory of change.

Outcomes: 19 thematic areas of interest as proposed by the World Health Organization with input from a technical advisory group (see Table 1).

**Table 1.** Definitions of thematic areas of interest.

**Entry stage**	
Data & evidence	Data collection and evaluation methods, knowledge mobilization, and mitigation of its absence
Needs identification	Needs that the health care workforce addresses, needs of the care workforce, and needs for integration of the workforces
Governance	Governance mechanisms, requirements, and jurisdiction effects
Funding	Funding allocations, challenges, transparency, and accountability
Pre-service education	Educational requirements and how they can be supported. Accreditation of educational bodies and governance of accreditation. Integration with health worker educational system.
Recruitment	Planning for and meeting dynamic needs and identify potential linkages with health workforce recruitment
**Working/Supportive stage**	
Professional regulation	Extent and approach to regulation of the care sector, and governance pathways for regulatory regimes
Organizational leadership	Management strategies to ensure adequate motivation, retention, support, supervision, and leadership for an integrated health and care workforce
Working conditions	Assurance of safe, healthy, and fair working conditions with respect to employers and care recipients
Scope of practice	Balance of skills, and range of services provided by care workers and managing overlaps with health workers
Support systems	Support system requirements, adaptations, and optimizations for an integrated workforce, including areas of technology and supply chains
Compensation & benefits	Determination of remuneration and mechanisms for implementation, funding considerations and inclusion of voluntary carers
Life-long learning	Skills to be reinforced or added over the career of care workers, and methods of doing so
Career advancement	Pathways for care workers to greater responsibility, higher positions, and higher earnings, and linking them to those pathways in the health workforce
Community integration	Acceptance, embeddedness, support, and challenges at the local community and household level, including rural and remote populations
**Exit stage**	
Retention/retirement	Impact of other themes and policies on enhancement of workforce retention and mitigation of the effects of retirement
Career choice/career pathways	Options and supports for workers leaving care work, possibly for health work
**Cross-cutting themes**	
Occupational segregation & equity	Occupational segregation within the care workforce and with respect to the health workforce, especially regarding gender, race, ethnicity, and immigration, and policies addressing inequities in compensation, benefits, working conditions, and advancement
Public & private ownership	Differences between public and private employment and participation in the care labor market

The outcomes are grouped according to the working lifespan model of human resources for health, which specifies 3 stages of workforce management—entry, working, and exit.^17^

### Search processes, data extraction, and quality assessment

Led by an academic librarian (Author Linton), we first piloted our search strings (see Appendix A2) (To access the Appendix, click on the Details tab of the article online) and then searched CINAHL on EBSCOhost, Medline, EMBASE, Scopus, and CAB International's Global Health database on the OVID platform using English search terms. Searches occurred in October 2023, March 2024, and November 2024. Gray literature was identified through manual Google advanced searches from June to July 2024 (see Appendix A3).

A team of 5 screeners (authors 1 through 5) were paired randomly and independently reviewed titles and abstracts of all sources using pre-determined inclusion and exclusion criteria (see Appendix A4). A third screener resolved discrepancies, or they were discussed at consensus meetings. A data extraction form was used to gather descriptive information and the CMO configuration according to the thematic areas of interest (see Appendix A5). Source quality was assessed using the Quality Assessment for Diverse Studies (QuADS) tool.^18^

## Results

The searches generated 7396 peer-reviewed sources and 481 gray literature sources. After all necessary steps of review, 138 academic and 13 gray literature sources were included for a total of 151 sources (see Appendix A6 for the PRISMA flow diagram). Twenty-two abstracts in other languages (eg, French, Spanish, and Japanese) were translated with Google Translate and full texts using DeepL Translator, a machine translation service, but these sources did not meet the inclusion criteria. The overall quality of the included sources was good, with a median QuADS score of 30 out of 39 (see Appendix A7). Source methods were quantitative (36%, *n* = 55), qualitative (30%, *n* = 45), mixed (23%, a *n* = 34), and “other” (11%, *n* = 17) such as policy analyses (see Appendix A8). The interventions took place in 46 different countries, with 5 countries as the context of 65% the sources (United States, United Kingdom, Australia, Canada, and the Netherlands, see Appendix A9).

### Findings

We refer to specific articles by source number as indicated in the citation list—Appendix A10. Most of the interventions were highly complex and reported on multiple outcomes (see Appendix A11). Below, we discuss the outcomes reported in the sources organized by the 3 stages of the working lifespan framework and the cross-cutting themes (see Table 2). Table 3 provides a detailed overview of all sources included in this review, including the source number, quality score, a description of each intervention, and its CMO configuration. When reading the findings, high-quality sources with QuADS scores in the top 2 quartiles are indicated with an asterisk.

**Table 2.** Sources by thematic areas of interest.

Thematic area	Source			
Entry				
**Data & evidence**	S4 Australia 2021	S85 Morgan 2018	S120 Squillace 2009	S139 Wilkinson 2021
	S46 Fischer 2020	S91 Navarra 2023	S134 VerValin 2018	S150 SfC 2024
**Needs identification**	S43 Elbourne 2015	S60 Hjelm 2000	S119 Sogstad 2020	
	S53 Håland 2021	S108 Rödlach 2009	S151 Varese 2024	
**Governance**	S4 Australia 2021	S46 Fischer 2020	S85 Morgan 2018	S119 Sogstad 2020
	S10 Bernard 2005	S53 Håland 2021	S86 Mun 2023	S121 Suter 2017
	S22 Chen 2020	S56 Hanlon 2007	S87 Murphy 2022	S122 Syson 2018
	S23 Chester 2014	S58 Harlock 2020	S90 Nandram 2014	S123 Szczepura 2023
	S25 Christensen 2017	S64 Kemper 2008	S91 Navarra 2023	S128 Tyler 2022
	S26 Clapper 2023	S65 Khavjou 2024	S97 Øvretveit 2010	S129 Udesen 2021
	S38 Dix 2023	S66 Kim 2019	S108 Rödlach 2009	S139 Wilkinson 2021
	S43 Elbourne 2015	S69 Kubo 2014	S110 Russell 2022	S143 Yan 2023
Funding	S6 Barnett 2018	S46 Fischer 2020	S92 Norman 2018	S119 Sogstad 2020
	S14 Boult 2011	S56 Hanlon 2007	S96 Ornstein 2011	S128 Tyler 2022
	S18 Burgdorf 2020	S58 Harlock 2020	S97 Øvretveit 2010	S134 VerValin 2018
	S22 Chen 2020	S69 Kubo 2014	S108 Rödlach 2009	S139 Wilkinson 2021
	S30 Craswell 2020	S83 Monro 2021	S110 Russell 2022	S150 SfC 2024
	S39 Douglas 2023	S86 Mun 2023	S117 Smith-Carrier 2015	S151 Varese 2024
	S42 Edes 2014	S90 Nandram 2014		
**Pre-service education**	S2 Arain	S50 Gaber 2020	S87 Murphy 2022	S108 Rödlach 2009
	S6 Barnett 2018	S68 Kroezen 2018	S92 Norman 2018	S112 Savassi 2021
	S7 Basnight 2023	S69 Kubo 2014	S95 Ochylski 2017	S115 Smith 2013
	S21 Chen 2014	S73 Lawlis 2016	S98 Pagaiya 2021	S118 Smyth 2015
	S33 Deshong 2010	S77 LTSS 2018	S99 Parveen 2021	S119 Sogstad 2020
	S44 Feldman 2019	S83 Monro 2021	S102 Poulain 2023	S128 Tyler 2022
	S46 Fischer 2020	S85 Morgan 2018	S105 Robertson 2023	S150 Varese 2024
**Recruitment**	S7 Basnight 2023	S37 Dill 2022	S87 Murphy 2022	S128 Tyler 2022
	S10 Bernard 2005	S46 Fischer 2020	S92 Norman 2018	S130 van der Borg 2017
	S18 Burgdorf 2020	S64 Kemper 2008	S98 Pagaiya 2021	S137 Wilberforce 2023
	S19 Chapman 2023	S69 Kubo 2014	S102 Poulain 2023	S142 Wu 2021
	S21 Chen 2014	S74 Lea 2023	S106 Robyn 2015	S143 Yan 2023
	S22 Chen 2020	S77 LTSS 2018	S108 Rödlach 2009	S150 SfC 2024
	S23 Chester 2014	S81 Merkel 2019	S110 Russell 2022	
	S25 Christensen 2017	S85 Morgan 2018	S112 Savassi 2021	
**Professional Regulation**	S1 Aldeghi 2013	S47 Florek 2022	S74 Lea 2023	S87 Murphy 2022
	S14 Boult 2011	S71 Lacher 2015	S85 Morgan 2018	S138 Wild 2011
**Organizational leadership**	S3 Ashwood 2016	S53 Håland 2021	S84 Morgan 2008	S116 Smith 2017
	S6 Barnett 2018	S54 Hald 2021	S86 Mun 2023	S117 Smith-Carrier 2015
	S9 Bayly 2018	S57 Hanssen 2017	S90 Nandram 2014	S122 Syson 2018
	S11 Bjerregaard 2015	S58 Harlock 2020	S96 Ornstein 2011	S124 Temkin-Greener 2020
	S14 Boult 2011	S59 Hjelle 2016	S97 Øvretveit 2010	S125 Tsui 2022
	S15 Boumans 2008	S61 Hollinger-Smith 2001	S99 Parveen 2021	S128 Tyler 2022
	S19 Chapman 2023	S64 Kemper 2008	S107 Rodgers 2017	S130 van der Borg 2017
	S34 Devine 2006	S68 Kroezen 2018	S109 Røsstad 2017	S131 van der Kooij 2013
	S25 Dill 2010	S70 Kulnik 2017	S110 Russell 2022	S139 Wilkinson 2021
	S39 Douglas 2023	S72 Larsson 2014	S113 Schoville 2020	S140 Woodward 2023
	S43 Elbourne 2015	S80 McDaniel 2011	S114 Sexton 2021	
	S50 Gaber 2020		S115 Smith 2013	
**Working conditions**	S15 Boumans 2008	S72 Larsson 2014	S106 Robyn 2015	S130 van der Borg 2017
	S16 Brown 2016	S74 Lea 2023	S108 Rödlach 2009	S131 van der Kooij 2013
	S21 Chen 2014	S80 McDaniel 2011	S110 Russell 2022	S132 van Haeften-van Dijk 2017
	S38 Dix 2023	S81 Merkel 2019	S114 Sexton 2021	S133 van Weert 2005
	S39 Douglas 2023	S83 Monro 2021	S117 Smith-Carrier 2015	S136 Warmoth 2023
	S43 Elbourne 2015	S84 Morgan 2008	S124 Temkin-Greener 2020	S141 Woolrych 2013
	S44 Feldman 2019	S87 Murphy 2022	S125 Tsui 2022	S142 Wu 2021
	S46 Fischer 2020	S88 Naccarella 2018	S126 Tullar 2016	S146 Crevacore 2024
	S52 Guerrero 2020	S90 Nandram 2014	S127 Tveito 2009	S147 Kelly 2024
	S59 Hjelle 2016	S91 Navarra 2023	S128 Tyler 2022	S148 McKay 2024
	S63 Hult 2023	S98 Pagaiya 2021	S129 Udesen 2021	S150 SfC 2024
	S67 Kornas 2021	S99 Parveen 2021		
**Working/support**				
**Scope of practice**	S2 Arain 2017	S38 Dix 2023	S83 Monro 2021	S119 Sogstad 2020
	S3 Ashwood 2016	S39 Douglas 2023	S90 Nandram 2014	S121 Suter 2017
	S4 Australia 2021	S41 Dryden 2009	S93 Obayashi 2020	S122 Syson 2018
	S7 Basnight 2023	S50 Gaber 2020	S94 Ochieng 2022	S124 Temkin-Greener 2020
	S15 Boumans 2008	S51 Gleason 2024	S98 Pagaiya 2021	S126 Tullar 2016
	S17 Bunn 2020	S53 Håland 2021	S105 Roberson 2023	S129 Udesen 2021
	S26 Clapper 2023	S59 Hjelle 2016	S107 Rogers 2017	S133 van Weert 2005
	S27 Clarke 2019	S60 Hjelm 2000	S111 Sandoz 2019	S136 Warmoth 2023
	S30 Craswell 2020	S74 Lea 2023	S113 Schoville 2020	S146 Crevacore 2024
	S32 Denton 2015	S75 Lee 2015	S117 Smith-Carrier 2015	S149 Roth 2024
	S33 Deshong 2010	S76 Lokmic-Tomkins 2021	S118 Smyth 2015	
**Support systems**	S3 Ashwood 2016	S57 Hamer 2018	S94 Ochieng 2022	S129 Udesen 2021
	S4 Australia 2021	S59 Hjelle 2016	S96 Ornstein 2011	S130 van der Borg 2017
	S19 Chapman 2023	S60 Hjelm 2000	S104 Robben 2012	S133 van Weert 2005
	S29 Cook 2017	S72 Larsson 2014	S107 Rodgers 2017	S135 Warmoth 2022
	S31 DeGraves 2024	S79 Manheim 2021	S108 Rödlach 2009	S136 Warmoth 2023
	S40 Dreher 2019	S86 Mun 2023	S109 Røsstad 2017	S139 Wilkinson 2021
	S45 Finnema 2005	S89 Nadav 2021	S117 Smith-Carrier 2015	S140 Woodward 2023
	S46 Fischer 2020	S90 Nandram 2014	S122 Syson 2018	S141 Woolrych 2013
	S49 Franzosa 2023	S91 Navarra 2023	S125 Tsui 2022	S144 Young 2023
	S50 Gaber 2020	S92 Norman 2018	S127 Tveito 2009	S146 Crevacore 2024
	S51 Gleason 2024	S93 Obayashi 2020	S128 Tyler 2022	S151 Varese 2024
	S55 Håland 2021			
**Compensation & benefits**	S4 Australia 2021	S37 Dill 2022	S84 Morgan 2008	S126 Tullar 2016
	S8 Baughman 2010	S44 Feldman 2019	S85 Morgan 2018	S134 VerValin 2018
	S11 Bjerregaard 2015	S46 Fischer 2020	S87 Murphy 2022	S141 Woolrych 2013
	S20 Charlesworth 2024	S62 Huang 2020	S91 Navarra 2023	S142 Wu 2021
	S21 Chen 2014	S65 Khavjou 2024	S98 Pagaiya 2021	S143 Yan 2023
	S22 Chen 2020	S66 Kim 2019	S108 Rödlach 2009	S147 Kelly 2024
	S33 Deshong 2010	S71 Lacher 2015	S110 Russell 2022	S149 Roth 2024
	S35 Dill 2010	S74 Lea 2023	S125 Tsui 2022	S150 SfC 2024
	S36 Dill 2014			
**Life-long learning**	S3 Ashwood 2016	S39 Douglas 2023	S78 Luz 2015	S112 Savassi 2021
	S4 Australia 2021	S40 Dreher 2019	S81 Merkel 2019	S116 Smith 2017
	S6 Barnett 2018	S41 Dryden 2009	S82 Meyer 2018	S117 Smith-Carrier 2015
	S9 Bayly 2018	S43 Elbourne 2015	S83 Monro 2021	S118 Smyth 2015
	S11 Bjerregaard 2015	S44 Feldman 2019	S84 Morgan 2008	S122 Syson 2018
	S12 Boscart 2018	S46 Fischer 2020	S85 Morgan 2018	S131 van der Kooij 2013
	S13 Boscart 2019	S52 Guerrero 2020	S86 Mun 2023	S132 van Haeften-van Dijk 2017
	S19 Chapman 2023	S55 Hamer 2018	S87 Murphy 2022	S133 van Weert 2005
	S21 Chen 2014	S57 Hanssen 2017	S94 Ochieng 2022	S136 Warmoth 2023
	S24 Choy 2016	S60 Hjelm 2000	S99 Parveen 2021	S140 Woodward 2023
	S27 Clarke 2019	S67 Kornas 2021	S101 Pointu 2005	S141 Woolrych 2013
	S28 Coogle 2007	S68 Kroezen 2018	S103 Reymond 2005	S145 Zeilig 2015
	S33 Deshong 2010	S70 Kulnik 2017	S107 Rodgers 2017	
	S35 Dill 2010	S73 Lawlis 2016	S110 Russell 2022	
	S38 Dix 2023	S74 Lea 2023	S111 Sandoz 2019	
**Career advancement**	S1 Aldeghi 2013	S30 Craswell 2020	S50 Gaber 2020	S85 Morgan 2018
	S4 Australia 2021	S33 Deshong 2010	S51 Gleason 2024	S87 Murphy 2022
	S6 Barnett 2018	S36 Dill 2014	S68 Kroezen 2018	S110 Russell 2022
	S17 Bunn 2020	S37 Dill 2022	S75 Lee 2015	S142 Wu 2021
	S21 Chen 2014	S44 Feldman 2019	S77 LTSS 2018	S150 SfC 2024
	S24 Choy 2016	S46 Fischer 2020	S81 Merkel 2019	
**Community integration**	S4 Australia 2021	S50 Gaber 2020	S100 Paulus 2005	S117 Smith-Carrier 2015
	S9 Bayly 2018	S53 Håland 2021	S103 Reymond 2005	S129 Udesen 2021
	S34 Devine 2006	S79 Manheim 2021	S108 Rödlach 2009	
**Exit**				
**Retention/retirement**	S1 Aldeghi 2013	S28 Coogle 2007	S81 Merkel 2019	S126 Tullar 2016
	S3 Ashwood 2016	S35 Dill 2010	S84 Morgan 2008	S128 Tyler 2022
	S5 Bandini 2024	S40 Dreher 2019	S90 Nandram 2014	S142 Wu 2021
	S16 Brown 2016	S44 Feldman 2019	S92 Norman 2018	S143 Yan 2023
	S19 Chapman 2023	S62 Huang 2020	S110 Russell 2022	S149 Roth 2024
	S21 Chen 2014	S80 McDaniel 2011	S116 Smith 2017	S150 SfC 2024
	S23 Chester 2014			
**Career choice/career pathways**	S4 Australia 2021	S71 Lacher 2015	S126 Tullar 2016	
	S37 Dill 2022	S76 Lokmic-Tomkins 2021	S142 Wu 2021	
**X-cutting**				
**Occupational segregation & equity**	S5 Bandini 2024	S25 Christensen 2017	S91 Navarra 2023	S147 Kelly 2024
	S20 Charlesworth 2024	S63 Hult 2023	S102 Poulain 2023	S150 SfC 2024
	S21 Chen 2014	S77 LTSS 2018	S108 Rödlach 2009	S151 Varese 2024
	S22 Chen 2020	S78 Luz 2015	S140 Woodward 2023	
		S87 Murphy 2022	S142 Wu 2021	
**Public & private ownership**	S20 Charlesworth 2024	S35 Dill 2010	S66 Kim 2019	S125 Tsui 2022
	S21 Chen 2014	S43 Elbourne 2015	S69 Kubo 2014	S150 SfC 2024
	S29 Cook 2017	S62 Huang 2020	S121 Suter 2017	

All sources are listed multiple times as they addressed more than one thematic area of interest. For more information, see Table A-ll in the Supplementary material.

**Table 3.** Detail of intervention and what worked, including study quality ranking by quartiles, context (C), mechanism (M), and working lifespan outcome (O)categories.

Source	Source ID	Quality	Intervention	Context			Mechanism	Outcomes	Working lifespan stage			
		(rank by quartiles)		country	scope	setting	action(s) and assumptions	What worked and for whom?	entry	support	exit	X-cut
Aldeghi 2013	S1	Q1	Accreditation for work experience	France	National	Community care	Allowing flexibility in certification supports retention efforts	Evaluating experience as part of credentialing helped retain experienced workers.	X	X	X	
Arain 2017	S2	Q3	Upskilling in administering medications	Canada	Regional	Residential care	Provide training to expand workforce capacity	Health care aides were more “fully used” in line with their training and competencies in lower-need residential care settings than high-need skilled-nursing care settings, especially in terms of medication support.	X	X	X	
Ashwood 2016	S3	Q1	Team-based leadership	UK	Organizational	Community care	Implement group leadership to enhance collaboration and improve workforce outcomes	Management by a team of 4 nurse leads improved recruitment and retention of care aides, improved the work satisfaction of both nurses and aides, and improved institutional memory/knowledge during turnover and succession.	X	X	X	
Australia 2021	S4	Q4	Policies to improve community care	Australia	National	Multi-setting	Implementing policy change can have unintended consequences	Remote monitoring reduced the volume of care activities while responding to emerging need, but there were concerns that an increasingly “on-demand” workforce would erode working conditions. Ongoing training is expected to be a key workforce enabler as long as it is linked to career progression.	X	X	X	
Bandini 2024	S5	Q1	Training in self-care, communication, and conflict management	USA	Organizational	Hospital	Provide training to improve workforce retention	The program did not improve retention as was expected. The authors recommend improving pay, hours and schedules to improve retention.			X	X
Barnett 2018	S6	Q1	Partnerships in education	Australia	Organizational	Multi-setting	Provide resources to establish partnerships between educators and service providers	Education was best offered in a series of sessions across multiple platforms, and mixed workforce groups helped break down professional barriers. Care workers as peer educators established career advancement potential. Momentum faded after funding ceased	X	X		
Basnight 2023	S7	Q3	Position of Patient Attendant Safety Aides (PASA)— a step below nurse's aide	USA	Organizational	Hospital	Establish a new care worker role to enhance workforce capacity	The “lay health worker” reduced the workload of higher paid staff and created a career pathway below nurses' aide	X	X		
Baughman 2010	S8	Q3	Wage pass-through; funding directed at increasing wages	USA	National	Multi-setting	Implement wage-pass through programs to improve worker pay	Targeted funding for workforce compensation increased the average wage of workers, but effects varied depending on policy levers (mandatory or voluntary, fixed or proportional funding, and means of reporting and compliance)		X		
Bayly 2018	S9	Q3	Position of knowledge broker—leadership role in dementia care	Canada	Organizational	Community care	Develop a knowledge broker role to develop workforce capacity	Knowledge brokers effectively liaise across clients, care workers and health professionals, improving workforce knowledge and building capacity.		X		
Bernard 2005	S10	Q2	Registration of designated carers in adult placement	UK	National	Community care	Shift in onus from designated carer to organization for registration	Changes to registration were quickly implemented and increased the recruitment and retention of carers	X			
Bjerregaard 2015	S11	Q2	Workers earning additional qualifications	UK	National	Multi-setting	Increase pay and recognition to support retention	Gaining a qualification increases care workers' well-being and their attachment to their job.		X		
Boscart 2018	S12	Q1	Upskilling for clinical assessments	Canada	Organizational	Residential care	Provide training to develop workforce capacity	The program successfully enhanced nursing assistants' capacity to assess the needs of residents		X		
Boscart 2019	S13	Q3	Online training with English as a second language	Canada	Organizational	Community care	Provide accessible training to support recruitment	Online education was accessible and low cost, and significantly improved students' knowledge and skills.		X		
Boult 2011	S14	Q3	Coordination through a care plan	USA	Organizational	Community care	Establish a coordinator role to enhance collaboration	Team leadership by a care guide effectively coordinated care and reduced the use of health services	X	X		
Boumans 2008	S15	Q4	Integrated care	Netherlands	Organizational	Residential care	Implement integrated care model to optimize the skill mix	Integrated care increased collaboration and reduced job demands, but there were no significant changes in job satisfaction		X		
Brown 2016	S16	Q3	The Green House model—an alternative residential care environment	USA	Organizational	Residential care	Use Green House model in residential care to improve retention	The organizational culture of the Green House model promotes staff longevity and retention		X	X	
Bunn 2020	S17	Q1	Position of diabetes specialist for health care aides	UK	Regional	Community care	Establish specialized care worker role to develop workforce capacity	Specialized care aides perform a unique role and have higher prestige than non-specialized aides in this study, representing an opportunity for career advancement		X		
Burgdorf 2020	S18	Q2	Expanding coverage for community care	USA	Regional	Community care	Increase resourcing to expand services	Expanded coverage was financially feasible and led to growth of the care workforce	X			
Chapman 2023	S19	Q1	New technologies	USA	Multi-level	Multi-setting	Experiment with new technologies to address workforce issues	Technology such as robots and remote monitoring do not replace workers but can reduce workloads. Communication and management systems improve scheduling and coordination across teams and systems and can enhance worker satisfaction. Online platforms are highly useful for recruitment.	X	X	X	
Charlesworth 2024	S20	Q3	International policies targeting the long-term care workforce	Multi	National	Multi-setting	Identify positive reforms in policy and practice to support the long-term care workforce	Working conditions are a key quality indicator for long-term care. This report includes an example of wage leveling to regularize worker pay across public and private sectors	X	X		X
Chen 2014	S21	Q3	Recruitment and retention initiatives	Multi	National	Multi-setting	Apply policy change in multiple policy arenas to enhance recruitment and retention	Recruitment and retention require collaboration across sectors and multi-pronged approaches: key factors include coherence between care and labor policies, compensation and working conditions (with a focus on migrant workers), education, and career development	X	X	X	X
Chen 2020	S22	Q1	Developing community care services	Taiwan	National	Multi-setting	Introduce transformative policy reform to develop the long-term care sector	Using multi-pronged complex policy levers, Taiwan is an example of the successful expansion of long-term care coverage within a short time period	X	X		X
Chester 2014	S23	Q3	Contracts with private and nonprofit services	UK	National	Multi-setting	Change contracting process to support retention	Contracting processes (“spot contracts” payment by use or “block contracts” payment for a fixed quantity) had no significant effect on the workforce; training is identified as a key factor influencing the quality and retention of workers	X		X	
Choy 2016	S24	Q2	Education programs for care workers	Australia	National	Residential care	Assess learning preferences to enhance accessibility and develop capacity	Study concluded that practice-based continuing education is best suited to the care workforce		X		
Christensen 2017	S25	Q4	Immigration and labor policies	Multi	International	Multi-setting	Identify motivating factors for health and care worker immigration	Based on data from the UK and Norway, foreign-born workers choose a destination country based on the availability of jobs, ease-of-entry, working conditions and protections, plus their perceptions of additional opportunities	X			X
Clapper 2023	S26	Q1	Skill mix in care teams	Netherlands	Organizational	Community care	Change team composition to reduce costs	More optimal team composition can be practically achieved by increasing integration between teams, or by applying more radical team redesign.	X	X		
Clarke 2019	S27	Q1	Upskilling for clinical assessments	UK	Organizational	Community care	Provide training to develop workforce capacity	Upskilling in clinical assessments was successful		X		
Coogle 2007	S28	Q2	Geriatric case management training program	USA	Organizational	Community care	Provide training to enhance retention	Better capacities in communication, problem-solving, and stress management improved the job satisfaction and career commitment of care aides		X	X	
Cook 2017	S29	Q3	Integration of health and social care services	UK	Regional	Residential care	Implement communication system to enhance collaboration	An example of a service model for residential care that enables information exchange and cooperation across organizational and professional boundaries		X		X
Craswell 2020	S30	Q3	Acute care in residential care facilities	Australia	Organizational	Residential care	Establish a coordinator role to enhance collaboration	Improved information sharing with physicians and hospitals improved health outcomes for residents and reduced length of hospital stays	X	X		
DeGraves 2024	S31	Q3	Training in “coherent breathing”	Canada	Organizational	Residential care	Provide training to support worker well-being	Training resulted in improvements in stress, psychological distress, and other mental health outcomes.		X		
Denton 2015	S32	Q4	Changing workforce skill mix through task shifting	Canada	Organizational	Community care	Develop the capacity of care staff to reduce costs	Task shifting to lower wage workers reduces labor costs but requires training and should be accompanied by increased pay		X		
Deshong 2010	S33	Q1	Position of Assistant in Nursing	Australia	Organizational	Hospital	Establish a new care worker role to enhance workforce capacity	New role of “assistant in nursing” was a low-cost option to respond to a shortage of trained nurses. In-house training was successful and retention was acceptable, with many going on to higher-level nursing education	X	X		
Devine 2006	S34	Q1	Coordination through a care plan	UK	Organizational	Multi-setting	Implement health action plans to support person-centred care	Nurses are best positioned to act as facilitators and coordinators across health and social services		X		X
Dill 2010	S35	Q4	Training in problem-solving, communication, and stress management	USA	Regional	Residential care	Implement a tiered training program with financial rewards	Career ladders in the form of educational opportunities accompanied by increased pay and recognition reduced staff turnover		X	X	
Dill 2014	S36	Q4	Linking compensation and education—Win a Step-Up program	USA	Regional	Multi-setting	Establish paths for promotion to support retention	Career ladders can be established by partnerships between service delivery and educational organizations, but only if accompanied by increased compensation and recognition		X		
Dill 2022	S37	Q4	Certifications among nonprofessional and allied health occupations	USA	National	Multi-setting	Establish certification to improve working conditions	Occupations with certifications lead to better job opportunities and higher compensation, and this is significantly enhanced by unionization	X	X	X	
Dix 2023	S38	Q2	Rapid training in the context of the pandemic	Australia	National	Residential care	Implement rapid training to expand workforce capacity	This study demonstrates a way to quickly expand scope of practice—implementation of rapid training in the use of personal protective equipment was supported by partnerships between service providers and the educational sector	X	X		
Douglas 2023	S39	Q3	Communication for dementia care	USA	Organizational	Residential care	Train in communication for people with dementia	Training by speech pathologists was highly effective for improving staff communication with residents with dementia, but funding for health professionals acting as instructors rather than clinicians was problematic in the American context.	X	X		
Dreher 2019	S40	Q3	Professional development—Win a Step-Up program	USA	Organizational	Residential care	Implement compassion fatigue awareness and self-care training	Brief training program was low-cost and well-received but inconclusive regarding retention		X	X	
Dryden 2009	S41	Q1	Exposure to palliative care	UK	Organizational	Community care	Provide training to develop workforce capacity	One-day training was effective for increasing care aides' knowledge of palliative care		X		
Edes 2014	S42	Q1	Home-based primary care (HBPC)	USA	National	Community care	Shift funding toward home-based primary care to expand services	Expanded community care services reduced overall health system costs, enhanced person-centred care and improved relationships with members of the integrated care team	X			
Elbourne 2015	S43	Q4	Bridging residential long-term care and community care	UK	Regional	Residential care	Establish intermediate care facilities to reduce system costs	Intermediate care reduces pressure on a variety of upstream and downstream services and is an example of multi-sectoral action in the UK. Implementation was “often fraught” and staff underwent a variety of “tribulations” but the project was ultimately successful and produced positive outcomes for service users	X	X		X
Feldman 2019	S44	Q4	Education incorporating peer mentoring	USA	Organizational	Community care	Introduce peer mentoring to improve retention	Homecare Aide Workforce Initiative (HAWI) incorporating peer mentoring had a positive influence on turnover	X	X	X	
Finnema 2005	S45	Q4	Emotion-oriented dementia care	Netherlands	Organizational	Residential care	Provide training to develop workforce capacity	Emotion-oriented care had a positive effect on many residents and reduced stress for some staff		X		
Fischer 2020	S46	Q4	Workforce strengthening strategies	USA	National	Multi-setting	Identify promising practices to strengthen the care workforce	Study identifies several opportunities to strengthen the care workforce including national job descriptions, value-based payments rather than fee-for-service, low, or no-cost training, and increasing wages.	X	X		
Florek 2022	S47	Q1	Health care aide registration schemes	Multi	National	Multi-setting	Register health care aides to support workforce management	Comparing registration schemes across 5 countries, regulation helps support workforce planning. Authors recommend the involvement of workers in planning, no-fee registration, and sufficient resourcing	X			
Fong 2022	S48	Q4	Training programs tied to quality-improvement measures	USA	Regional	Community care	Provide training to develop workforce capacity	Quality-improvement training had the most benefit for high-need care recipients but had inconclusive workforce outcomes		X		
Franzosa 2023	S49	Q2	Health and care services for veterans	USA	National	Community care	Enhance collaboration to improve health outcomes	Health care is improved by better integration of care aides; this involves incentivizing the coordination between medical care and long-term care and removing communication barriers between medical professionals and care workers		X		
Gaber 2020	S50	Q3	Volunteers collecting health information	Canada	Regional	Community care	Equip and train community volunteers to collect health information	Home visits by volunteers equipped with eHealth technologies were highly effective for collecting health information and supporting clients, but the study identified low integration with medical professionals.	X	X		
Gleason 2024	S51	Q2	Mentoring program	USA	Organizational	Residential care	Implement tele-mentoring by nurses to support the care workforce	Training nurse leaders and implementing the mentoring program improved care aide's self-efficacy, enhanced their skills, and reduced feelings of professional isolation		X		
Guerrero 2020	S52	Q2	Dementia care training in community care	USA	Organizational	Community care	Train paid and unpaid caregivers to develop capacity	Training generally increased knowledge of dementia care but authors suggest considering unique need of subpopulations of unpaid caregivers and assessing prior knowledge among experienced care workers		X		
Håland 2015	S53	Q1	Transitions from hospital to home	Norway	Regional	Multi-setting	Establish procedures to support person-centred care	Patient Trajectory for Home-dwelling elders (PaTH) procedures improved communication across organizational boundaries, but hospitals tended to be resistant to change. Implementation was smoother when leaders were responsive to the concerns of workers	X	X		
Hald 2021	S54	Q3	Integration of health and social services	Denmark	Regional	Multi-setting	Implement integrated care model to improve health outcomes	Implementing integration and enhancing interprofessional collaboration requires attention to contextual factors		X		
Hamer 2018	S55	Q1	Quality-improvement	UK	Organizational	Hospital	Provide training to develop workforce capacity	Nurse-led training increased care aide's confidence performing tasks they were previously unsure about, and improved communication between aides and nursing staff		X		
Hanlon 2007	S56	Q1	Regulation of voluntary and nonprofit services	Canada	Regional	Community care	Introduce policies to encourage market competition and monitor community care sector	Emphasis on efficiency and competition pressured small nonprofits to close down or consolidate into larger organizational entities, eroding service flexibility and personalization	X			
Hanssen 2017	S57	Q4	Training in collaboration with informal caregivers	Norway	Regional	Multi-setting	Provide training to improve collaboration with unpaid carers	Online training was cost-effective and improved collaboration with informal carers. Enrollment was strongly influenced by organizational leadership; a certification process would enhance uptake.		X		
Harlock 2020	S58	Q2	Pooled budgets for health and social care	UK	Regional	Multi-setting	Pool funding to promote integration across health and care services	Pooling budgets across health and social care sectors enforces collaboration but leadership is essential to mitigate conflict. A culture of trust, willingness to share information and the avoidance of “blame” are critical	X	X		
Hjelle 2016	S59	Q2	Reablement philosophy	Norway	National	Community care	Provide training to develop collaboration and workforce capacity	The reablement philosophy required integration and skill-transfer from health professionals to care workers, facilitated by spaces and schedules allowing for frequent team meetings. Upskilling occurred on-the-job rather than as a distinct educational intervention, with ongoing implications for staff turnover and new care workers untrained in the approach		X		
Hjelm 2000	S60	Q1	National standards for wound care	Sweden	Regional	Multi-setting	Assess workforce capacity regarding standards of care	Most care workers did not have proper training, although there was evidence of more training for nurses' aides in residential care settings	X	X		
Hollinger-Smith 2001	S61	Q1	Leadership training for nurses	USA	Organizational	Residential care	Provide leadership training to improve working conditions	The training program encouraged leadership qualities associated with empowerment and support for care staff, resulting in greater work effectiveness, less job stress, and greater job satisfaction		X		X
Huang 2020	S62	Q2	Ownership models in for-profit residential care	USA	Regional	Residential care	Ownership model affects worker retention	In the context of for-profit residential care, owner-managed facilities tend to have a better work culture and better retention of care workers		X	X	X
Hult 2023	S63	Q3	Increased on-call, part-time and temporary positions in the care workforce	Finland	National	Multi-setting	Increase flexibility in the care workforce to reduce costs	Precarious employment includes temporariness, low wages, disempowerment, vulnerability, fewer rights and lower exercise of rights. Precarity, highly apparent in the care sector, has a negative effect on mental and physical health and should be addressed		X		X
Kemper 2008	S64	Q2	Recruitment and retention initiatives	USA	Regional	Multi-setting	Provide training and pay increases to reduce turnover and improve recruitment	Key factors for the successful implementation of the “Better Jobs Better Care” project included sufficient resources, cross-sectoral partnerships, a neutral organization for overall leadership, stability of partnering organizations, and clearly defined goals	X	X		
Khavjou 2024	S65	Q3	Wage pass-through; service funding directed at increasing wages	USA	Regional	Multi-setting	Assess success of state policies aimed to increase worker pay	Wages increased in states implementing any type of wage pass through policy, but increases were small and care workers continue to make less than other entry-level occupations	X	X		
Kim 2019	S66	Q2	Minimum wage laws and worker's rights policies	USA	Regional	Multi-setting	Assess interactions between policy arenas to support wage increases	Generous minimum wage laws enacted in tandem with the Domestic Workers' Bill of Rights increased the wages of care workers across for-profit and nonprofit sectors, although the wages of those employed by individual households were not influenced by state policies. When policies targeting wages or workers' rights were enacted alone, worker pay was not improved	X	X		X
Kornas 2021	S67	Q3	Clinical services in community care	Canada	Organizational	Hospital	Develop role of care lead role to enhance collaboration and teamwork	Hospital-based care leads played an essential role in supporting information exchange and responding to concerns of community care providers.		X		
Kroezen 2018	S68	Q3	Educational curricula for care workers	Multi	International	Multi-setting	Assess educational frameworks to improve training	Study identified formal educational frameworks tied to regulation and certification, along with theory-based curriculum, result in higher levels of learning outcomes among the care workforce	X	X		
Kubo 2014	S69	Q2	National long-term care insurance	Japan	National	Community care	Establish a national insurance scheme for long-term care	Effective at growing the private care market, with swift increases in the number of care providers especially for-profit providers in large urban centers. The policy reform may have exacerbated workforce issues including low wages	X			X
Kulnik 2017	S70	Q3	Interprofessional training	UK	Regional	Community care	Provide interprofessional training to enhance collaboration	This project shows how multi-agency partnerships can benefit from interprofessional training, and how interprofessional training supports integration and facilitates collaboration across organizations		X		
Lacher 2015	S71	Q2	Position of Level 3 Allied Healthcare Assistant (AHA)	Switzerland	National	Hospital	Establish a new care worker role to grow the workforce	Allied Healthcare Assistant represents a new skill level between nurse's aides and registered nurse, creates a career ladder for care workers, increases competencies among care workers, and addresses nursing shortages	X	X	X	
Larsson 2014	S72	Q4	Workplace health promotion measures	Sweden	Organizational	Community care	Apply health promotion measures to support the workforce	Organizational and individual health promotion measures are both important in designing healthy workplaces		X		
Lawlis 2016	S73	Q3	Interprofessional educational program	Australia	Organizational	Residential care	Provide integrated education to support workforce integration	The pilot project in interprofessional education was found to enhance student learning and increase understanding of care for older adults	X	X		
Lea 2023	S74	Q3	Education for position of dementia care worker	Australia	Organizational	Multi-setting	Establish a new care worker role to develop workforce capacity	Peer-to-peer knowledge transfer improved learning and established a new career pathway, and participation was enhanced by providing a course fee waiver and wage increase on completion. The new position bridges gaps in the care workforce and provides an optimal skill mix for an aging population	X	X		
Lee 2015	S75	Q2	Upskilling for administering medications	Australia	Organizational	Community care	Expand the role of the care workforce to reduce costs	Care workers can safely administer basic medications a lower cost than nurses		X		
Lokmic-Tomkins 2021	S76	Q2	Education for position of health assistant in nursing (HAN)	Australia	National	Multi-setting	Employ nursing students as health care aides to improve training and expand care workforce capacity	The new position represents a career ladder between untrained care work and nursing with paid practical training, however, the HAN scope of practice may not support advancement through practical training		X	X	
LTSS 2018	S77	Q1	National and organizational policies related to foreign-born workers	Multi	Multi-level	Multi-setting	Provide more support for foreign-born workers to grow the care workforce	Countries can attract and retain foreign-born workers by increasing protections and improving worker rights. Employers can attract and retain foreign-born workers by valuing employee diversity, creating flexibility in scheduling, and providing effective employee evaluations	X			X
Luz 2015	S78	Q1	Personal Home Care Aide state training demonstration project	USA	Regional	Multi-setting	Provide accessible training to improve retention	Retention in training programs improves when programs are designed in consideration of the socio-economic realities of workers		X		X
Manheim 2021	S79	Q1	Home-based primary care (HBPC)	USA	National	Community care	Improve relationships to enhance retention	Relationship building and collaboration with unpaid caregivers improved staff retention		X	X	
McDaniel 2011	S80	Q3	Ethics environment as part of organizational culture	USA	Organizational	Residential care	Change work culture to improve retention	Changing work culture to have a strong ethics environment improved working conditions and staff retention	X	X	X	
Merkel 2019	S81	Q2	Age management for retention	Multi	National	Multi-setting	Implement age management practices to improve retention of older workers	Focusing on ageing management among staff, including flexible hours, reduced working hours, and health promotion can help address workforce shortages	X	X	X	
Meyer 2018	S82	Q1	Diversity training	Australia	Organizational	Community care	Implement 1/2 day diversity training	Adult learning principles were key to training, but the half-day workshop was too brief, and combined training of workers and managers was challenging		X		
Monro 2021	S83	Q2	Expanding coverage for community care	Australia	National	Residential care	Shift resourcing in the long-term care sector to enhance community-based services	Increased resourcing to community care resulted in reduced resourcing to residential care. There was also a change in resident characteristics to higher complexity. Existing training for care workers was insufficient to respond to higher care need, and organizations were left responsible for training and upskilling. There was also higher staff turnover and increased outsourcing to temp workers	X	X		
Morgan 2008	S84	Q2	Education tied to compensation—Win a Step-Up program	USA	Regional	Residential care	Implement tiered training program with financial rewards	The Win-a-Step-Up Program increased staff morale and job satisfaction, with modest reductions in turnover		X	X	
Morgan 2018	S85	Q2	State-level training models for care aides	USA	Regional	Multi-setting	Provide accessible training to help grow the workforce	Facilitators to program completion included remedial courses prior to training, in-person training, and multi-language course materials, and collaborations between industry and educational partners	X	X		
Mun 2023	S86	Q1	Transitions from hospital to home	South Korea	National	Multi-setting	Establish a coordinator role to enhance collaboration	Community care centers supported team building and helped revitalize home health care; case management, communication technology, and educational components were important to success, but hospitals require more incentivization	X	X		
Murphy 2022	S87	Q1	Recruitment and retention initiatives	Multi	National	Multi-setting	Assess strategies to strengthen the care workforce	Legislation aimed at improving worker protection supports recruitment and retention. Germany provides an example of worker protections extending to private homes	X	X		X
Naccarella 2018	S88	Q4	Environmental workplace design	Australia	Organizational	Residential care	Follow environmental design principles to support resident and staff well-being	Environmental workplace design (home-like environment with attention to factors such as comfort, lighting, plants) supports the well-being of care recipients and care workers		X		
Nadav 2021	S89	Q4	Digital communication system	Finland	National	Multi-setting	Provide communication system to enhance collaboration	New digital services were highly useful, but implementation was challenging. Study recommends a set of best practices for implementation		X		
Nandram 2014	S90	Q1	Buurtzorg Nederland model	Netherlands	Organizational	Community care	Implement Buurtzorg service delivery approach to improve working conditions and enhance retention	Buurtzorg is an example of an integrated multi-component approach delivering quality care and quality working environments with low overheads	X	X	X	
Navarra 2023	S91	Q1	The European Care Strategy (2022), a pan-European approach for member states.	Multi	International	Multi-setting	Implement recommendations to strengthen the care workforce	The European strategy recommends minimum wage and occupational safety directives, support for migrant and live-in domestic workers. and improvements to national occupational and industry definitions related to care	X	X		X
Norman 2018	S92	Q1	Home-based primary care (HBPC)	USA	National	Community care	Introduce home-based primary care involving care workers	Study highly recommends designing communication and scheduling technology specific to community-based mobile practice	X	X	X	
Obayashi 2020	S93	Q3	Communication robots	Japan	Organizational	Residential care	Use communication robots to reduce labor burden	Communication robots cannot replace workers but have the potential to reduce the workload of care workers in an assistive role		X		
Ochieng 2022	S94	Q4	Video consultations using virtual and augmented reality	UK	Organizational	Residential care	Implement video consultations to enhance collaboration	Physicians and workers found virtual consultations helped speed up treatment, but staff must be trained and confident in the use of the technology		X		
Ochylski 2017	S95	Q2	Personal Home Care Aide state training program	USA	Regional	Community care	Tailor educational programs to learner preferences	Most (60%) students preferred a combination of in-person and online training, but learners with lower income levels, education, and literacy skills preferred in-person training only	X			
Ornstein 2011	S96	Q1	Transitions from hospital to home	USA	Organizational	Multi-setting	Establish a coordinator role to enhance collaboration	Establishing a position of coordinator improved communication but did not decrease hospital stays. Funding the coordinator role was problematic in the American context	X	X		
Øvretveit 2010	S97	Q1	Pooled budgets for health and social care	Sweden	Multi-level	Multi-setting	Pool funding to improve integration between health and care services	A phased approach to implementation was necessary. Changes in the macro-structure of health and social funding gave managers the flexibility to improve coordination at the local level, but it required significant management capacity	X	X		
Pagaiya 2021	S98	Q3	Incorporating care workers in rural home health care teams	Thailand	Regional	Community care	Shift care workers to health worker role to expand services	In response to a shortage of professional health workers in rural community health care, care workers are a viable solution, but wages should be raised, and training may need to be increased	X	X		
Parveen 2021	S99	Q4	Dementia Training Standards framework	UK	National	Multi-setting	Provide training to develop workforce capacity	Although this educational intervention had limited success, courses combining in-person teaching and online simulations were most likely to have positive learning outcomes	X	X		
Paulus 2005	S100	Q2	Integrated care	Netherlands	Organizational	Residential care	Implement integrated care to enhance collaboration between staff and families	The introduction of integrated care caused little change to the balance between paid and unpaid care activities		X		
Pointu 2005	S101	Q1	Epilepsy awareness program	UK	Regional	Multi-setting	Provide training to develop workforce capacity	The program was highly successful, providing an example of a collaboratively developed training model focused on a specific health issue and population		X		
Poulain 2023	S102	Q1	Policies supporting the out-migration of care workers from India to Europe	India	International	Multi-setting	Assess worker protections in host countries to protect migrant health and care workers	India has established a number of country-to-country agreements to simplify the emigration of trained health professionals to European member states. The care sector has fewer labor protections and is considered less feasible	X			X
Reymond 2005	S103	Q1	Locally tailored workshops for training in palliative care	Australia	Regional	Community care	Provide training to develop workforce capacity	This educational intervention aimed at physicians and frontline workers was relatively inexpensive with reasonable reach and positive educational and clinical outcomes		X		
Robben 2012	S104	Q4	Digital communication system	Netherlands	Regional	Community care	Provide communication tool to enhance collaboration	Implementation was facilitated by the communication portal's usefulness and the involvement of workers and clients in its development, but there were challenges related to user-friendliness and computer literacy of users		X		
Robertson 2023	S105	Q2	Education for position of nursing assistant	UK	National	Multi-setting	Develop “nursing assistant” role between health care aide and registered nurse to develop workforce capacity	Nursing assistant's scope of practice was not clear, and some trainees had significant difficulties gaining required practical competencies as some can only be gained in hospital settings	X	X		
Robyn 2015	S106	Q4	Recruitment and retention in rural areas	Cameroon	Regional	Multi-setting	Provide financial incentives to grow the rural health and care workforce	Trainee nurses and nurses' aides were more willing to practice in rural areas if offered substantial financial bonuses and sufficient equipment and supplies	X	X		
Rodgers 2017	S107	Q1	Palliative care in residential care settings	New Zealand	Organizational	Residential care	Enhance collaboration to improve care	Collaboration with palliative care professionals helped residences re-align their philosophy, policies, and procedural guidelines in the context of palliative care. The characteristics of the collaboration varied depending on the needs and capacities of a given facility		X		
Rödlach 2009	S108	Q1	Trained voluntary caregivers	Zimbabwe	Regional	Community care	Draw on volunteer care workers to expand services	Volunteers can replace paid workers in the context of HIV patients, even for administering medicines and treatments. Supported by collaboration with community organizations, recruitment and retention of volunteers is enhanced by public recognition	X	X		X
Røsstad 2017	S109	Q2	Transitions from hospital to home	Norway	Regional	Multi-setting	Implement communication process to enhance collaboration	The introduction of checklists made little difference apart from more physician consultations		X		
Russell 2022	S110	Q3	Value-based payments and training programs	USA	Regional	Community care	Provide career advancement opportunities to enhance retention	WIOA (Workforce Intervention and Opportunity Act)—facilitated inter-sectoral collaboration; however, there was concern across agencies about competition for workers and clients. Training programs were effective but did not fully support the development of career ladders	X	X	X	
Sandoz 2019	S111	Q1	Training in wound care	UK	Regional	Community care	Provide training to enhance workforce capacity	Brief training programs support the upskilling of aides for delegated tasks such as wound care		X		
Savassi 2021	S112	Q4	Free online courses for health and care qualifications, a MOOC (Massive Open Online Course)	Brazil	National	Multi-setting	Provide free online training to grow the health and care workforce	Free online training portal covered all regions across the country, and there was a direct relationship between the number of course enrollments and the expansion of home health care programs	X	X		
Schoville 2020	S113	Q2	Communication technology	USA	Organizational	Residential care	Implement electronic health records to enhance collaboration	Information sharing across facilities improved workflow and workload, but staff need training in using new technology. It also added new duties for care assistants related to charting and administrative activities previously performed by nurses		X		
Sexton 2021	S114	Q4	Positive leadership walk rounds	USA	Organizational	Multi-setting	Implement positive leadership to improve working conditions	Positive leadership principles during walk rounds were associated with better well-being health and care workers		X		
Smith 2013	S115	Q1	Position of Level 3 certificate in aged care	Australia	National	Multi-setting	Establish a new care worker role to enhance workforce capacity	Training was highly inconsistent, employers were not satisfied with the skills and knowledge of graduates, and there was no consistency between job titles and roles of certified staff. The authors concluded that qualifications should be regulated and better defined	X	X		
Smith 2017	S116	Q4	Dementia training for care workers	UK	Organizational	Community care	Provide training to enhance workforce capacity	Dementia training increased worker confidence in understanding the needs of clients and families, with potential for increasing job satisfaction and improving retention		X	X	
Smith-Carrier 2015	S117	Q3	Home-based primary care (HBPC)	Canada	Organizational	Community care	Provide resources to enhance collaboration	Collaboration was facilitated by communication tools, positive leadership, and team-based learning, resulting in improved job satisfaction and better patient outcomes. Challenges included insufficient resources, professional power dynamics, hierarchical leadership, and unclear roles	X	X		
Smyth 2015	S118	Q1	Assessment tool for care workers	UK	Regional	Community care	Assess staff to support care quality	Assessment assured standards of care and reduced the time spent by nurses in training staff individually, but was stressful for workers	X	X		
Sogstad 2020	S119	Q4	“Care Plan 2015” expanding long-term care services	Norway	National	Multi-setting	Increase training and specialization to develop care workforce capacity	Specialization and level of education of care staff were higher in larger municipalities, and in municipalities with targeted revenues vs block grants. Dementia care and palliative care are high priority.	X	X		
Squillace 2009	S120	Q4	Data collection for the care workforce (National Nursing Assistant Survey)	USA	National	Residential care	Develop national data source on nursing assistants working in residential care	Allowed for linkages to existing health workforce data and better workforce planning	X			
Suter 2014	S121	Q3	Involving care workers in health care teams	Canada	Organizational	Residential care	Optimize the role of care staff to reduce pressure on nursing staff	Staff mix was negatively impacted by inconsistent roles and shifting responsibilities of care workers. For-profit facilities were more likely to use care staff for activities requiring higher levels of training, but training levels were inconsistent.	X	X		X
Syson 2010	S122	Q1	Health and social care teams	UK	Regional	Community care	Monitor team functioning to enhance collaboration	Integrated teams were supported by shared office space and information sharing processes. Results included better access, scheduling, and travel time. Implementation required flexibility and conflict resolution.	X	X		
Szczepura 2023	S123	Q2	Integrated long-term care “neighborhoods”	Multi	National	Multi-setting	Support innovation and new technologies to expand capacity	Japan has introduced “care science” as a new discipline to complement medicine and nursing. England is promoting pilot programs to assess various technological innovations to support the care workforce	X			
Temkin-Greener 2020	S124	Q4	Home-based primary care (HBPC)	USA	National	Community Care	Install nurses as team leaders to improve collaboration	Higher effectiveness was reported for teams led by nurses, with collaborative rather than hierarchical leadership, and with better resourcing and staffing levels		X		
Tsui 2022	S125	Q4	Supports for workers in the context of client death	USA	Organizational	Community Care	Implement supports and resources to support workforce well-being	Training in end-of-life care, peer support, and professional counseling services are recommended to support home care workers in managing the death of a care recipient		X		X
Tullar 2016	S126	Q4	Employee engagement program	USA	Organizational	Hospital	Improve employee engagement to enhance retention	Engagement program improved care worker retention		X	X	
Tveito 2009	S127	Q2	Health program for staff	Norway	Organizational	Residential Care	Provide training to support worker well-being	The program did not improve employee health but may improve job satisfaction		X		
Tyler 2022	S128	Q3	Mitigating staffing challenges during COVID-19	USA	Multi-level	Community Care	Provide career advancement opportunities to enhance retention	Policies that improved workforce retention included increased funding at the state level, in turn leading to increases at the organizational level in wages and benefits and the creation of within-agency career ladders for care workers	X	X	X	
Udesen 2021	S129	Q4	Acute-care teams for long-term care	Denmark	Regional	Multi-setting	Expand the capacity of long-term care staff to reduce hospital visits	Home-based acute-care teams reduced hospital visits (highly effective for intravenous therapy) and improved communication with physicians.	X	X		
van der Borg 2017	S130	Q4	Client-centred care initiative	Netherlands	Organizational	Residential care	Adopt client-centred care to improve health outcomes and working conditions	Client-centred care improved workplace culture and relationships, improving the overall well-being of workers.	X	X		
van der Kooij 2013	S131	Q2	Training in integrated emotion-oriented care for dementia	Netherlands	Regional	Residential Care	Provide training to enhance workforce capacity	Training improved dementia care quality without significantly impacting the amount of time spent on traditional care tasks		X		
van Haeften-van Dijk 2017	S132	Q4	Day centers	Netherlands	National	Community care	Provide day programs to support community-based dementia care	Meeting centers aimed at supporting carers and people with dementia also improved job satisfaction for care workers related to workspace		X		
van Weert 2005	S133	Q4	Non-verbal communication techniques for dementia care: Snoezelen (multi-sensory stimulation)	Netherlands	Organizational	Residential Care	Provide training to enhance workforce capacity	Resident affect was improved using non-verbal communication techniques with positive repercussions for worker experiences		X		
VerValin 2018	S134	Q3	Increasing wages in home care	USA	National/regional	Community Care	Increase wages to improve recruitment and retention	Investing in a living wage and providing better benefits for home health workers will improve recruitment and retention, with potential for reducing hospital costs and increasing consumption of this employment sector. Authors suggest a cost-benefit analysis is too simplistic, recommending future health care expenditure analyses be conducted through social welfare functions	X	X		
Warmoth 2022	S135	Q3	Video conferencing	UK	Organizational	Residential Care	Provide communication tool to enhance collaboration	Video conferencing was relatively easily implemented. The impact on staff was not assessed		X		
Warmoth 2023	S136	Q2	Video conferencing	UK	Organizational	Residential Care	Provide communication tool to enhance collaboration	Video conferencing allowed care workers to collaborate with and learn from health care professionals, and some reported reductions in their workload		X		
Wilberforce 2023	S137	Q3	Tool for evaluating prospective employees	UK	Organizational	Multi-setting	Assess care work applicants to hire those with strong interpersonal skills	Assessment tool was useful for hiring care workers with better interpersonal skills and judgement	X			
Wild 2011	S138	Q1	New vocational qualifications for direct care workers	UK	National	Multi-setting	Establish a care worker designation, develop a representative body, code of conduct, and clearly defined roles to enhance the status of care work	Care workers under the new designation felt positive about their professional status	X			
Wilkinson 2021	S139	Q2	Social prescribing	UK	Multi-level	Multi-setting	Implement social prescribing as part of service integration	This intervention started small (2 employees at a nonprofit organization plus volunteers), with increasing funding through collaboration across health and private/voluntary sector partners. Competition between community-based service providers tended to contribute to a lack of information sharing	X	X		
Woodward 2023	S140	Q2	Enhanced Health in Care Homes framework	UK	Regional	Multi-setting	Enhance collaboration to improve health outcomes	Collaboration improved information sharing between physicians and care workers, enhancing care workers' feelings of respect and raising their confidence in their ability to provide appropriate care		X		X
Woolrych 2013	S141	Q2	Integrated dementia care	UK	Regional	Community care	Enhance collaboration to improve health outcomes	Care workers need a clear role definition, flexibility in their work, more than minimum wage, and pathways to career progression		X		
Wu 2021	S142	Q3	Volume-based payments and minimum compensation	Taiwan	National	Community care	Introduce volume-based payments to enhance retention	The new payment model contributed to rapid growth in the care workforce, improving recruitment and retention. Flexibility in scheduling also increased worker's sense of autonomy	X	X	X	X
Yan 2023	S143	Q3	Minimum wage laws	USA	National	Community Care	Increase minimum wage to improve retention	Increasing minimum wage reduced the wage gap between the home health industry and other competing industries and will help retain home health aides	X	X	X	
Young 2023	S144	Q4	Online mindfulness training	USA	Organizational	Residential care	Provide training to support workforce well-being	Online mindfulness training was feasible and can help reduce depressive symptoms among nursing assistants		X		
Zeilig 2015	S145	Q1	Arts-based workshop for dementia care	UK	Organizational	Residential care	Improve the accessibility of training to enhance workforce capacity	An arts-based workshop offering an interactive mode of education effectively engaged care workers		X		
**Search Update: Sources added November 2024**												
Crevacore 2024	S146	Q3	Delegating nursing tasks to nursing assistants	Australia	Organizational	Hospital	Develop the capacity of care staff to reduce pressure on nursing staff	Fostering a clear understanding of the scope of practice of care workers and nurses supports task delegation		X		
Kelly 2024	S147	Q4	Cash for care programs	Canada	Regional	Community care	Position the care recipient as the employer and care coordinator to empower care recipients	Working directly for a client may provide more flexibility, autonomy and pay for workers. Working for an agency rather than directly for a client may provide more stability		X		X
McKay 2024	S148	Q2	Scheduling optimization through the Essential Care on Weekends (ECoW) program	Canada	Organizational	Community care	Change scheduling priorities to improve service delivery	New scheduling priorities improved working conditions for staff and increased service capacity and reliability		X		
Roth 2024	S149	Q3	Providing care services to homeless people	USA	Organizational	Community care	Implement context-specific supports to enhance retention	Staff retention was enhanced by context-specific training, flexibility in time off, and increased wages. Care workers “served as a bridge” improving connections with other health and social service providers		X	X	
SfC 2024	S150	Q2	Recruitment and retention strategies	UK	National	Multi-setting	Implement sector-wide strategies to support the workforce	Skills for Care provides a comprehensive set of free online resources for all employer types relating to many of the key thematic areas of interest, including effective recruitment and retention strategies, improving diversity and inclusivity, and protecting immigrant workers	X	X	X	X
Varese 2024	S151	Q4	Mental health service hubs for the health and care workforces	UK	National	Multi-setting	Establish mental health hubs to support the well-being of the health and care workforces	Mental health hubs are a flexible response to broad public crises ranging across war, conflict, climate change, and pandemics. Such hubs are an important resource to improve/support the overall well-being of the health and care workforces	X	X		

Q1 indicates sources ranked up to the 25th quartile, Q2 the 50th quartile, Q3 the 75th quartile, and Q4 the top quartile.

#### Entry stage: planning the care workforce

The entry stage of the working lifespan model involves planning for the care workforce, with interventions aimed at recruitment, governance, and pre-service education. There were few interventions addressing needs identification and the systematic collection of data and evidence.

Of the interventions solely focused on entry stage outcomes, most were concerned with governance and recruitment. One study assessing the introduction of long-term care insurance in Japan found it increased the volume of services and size of the care workforce but generated an oversupply of workers, leading to reduced wages (S69). As a response, the authors emphasized aligning training requirements with wages.

There was limited reporting on interventions related to data and evidence. For example, Squillace (S120*) described the development and evaluation of a National Nursing Assistant Survey in the United States for care workers in nursing homes, finding that a national survey such as this can improve knowledge about the workforce, link with existing health workforce datasets, and be used for care workforce planning and monitoring. The United Kingdom's sector-wide Skills for Care resources are built around an Adult Social Care Workforce Data Set (ASC-WDS), iteratively building capacity and monitoring the social care sector (S150). Developing shared occupational terminology and clear job descriptions is beneficial, especially in multi-country contexts such as the European Union (S91).

Pre-service education intersected with many other themes, representing a key lever for strengthening the care workforce. The interventions emphasized linkages between pre-service education and supportive on-the-job practices. The combination of consistent educational requirements plus central oversight was identified as essential for successful pre-service training interventions, and a necessary precursor for wage increases tied to specialization or higher training requirements (S115, S119*, S128*, and S150). Formalized pre-service educational programs in addition to clinical experiences are beneficial in most contexts (S68*, S98*, and S110*), and in-person or hybrid training is preferred by care workers (S85, S95). A scan of 28 European Member States found that theory-based curriculum improves learning outcomes and tends to be linked with formal educational frameworks and/or other forms of regulation (S68*). Low-cost or no-cost training opportunities further care worker recruitment and can support marginalized groups facing barriers to entry (S22, S37*, S46*, S85, S106*, S110*, S112*, and S128*).

Service providers are often involved with pre-service education. There were compelling examples of interprofessional education crossing the health and care, with partnerships between educational institutes and providers to enable field experience during training (S6, S73*, S80*, S105, and S118). For example, again in Australia, “Teaching and Research Aged Care Services” (TRACs) successfully fostered alliances and developed residential care partners as teaching sites for mixed workforce groups (S6). However, this initiative has since lost its funding.

There were limited interventions reporting on funding and governance in the sources in the review. Governance and funding interventions that aimed to expand or promote service integration had mixed findings. For example, in England, introducing new pooled funding through the Better Care Fund was challenging to implement, but enabled collaboration across workforces (S58). In contrast, a study assessing the care labor market in Australia (S4*) argues for a balance of “on-demand” system design with working conditions.

Many countries have policies to recruit immigrant or migrant care workers to fill labor shortages, and this trend may require a multi-jurisdictional or global approach (S25*, S77, S102, and S150). Foreign-born care workers tend to choose their destination country based on job prospects, working conditions, immigration policies, and their perceptions of additional opportunities (S25*). A qualitative study of international recruitment strategies in England, the Netherlands, and Taiwan emphasized the importance of alignment between labor laws, compensation, and working conditions (S21*). Recognizing the vulnerability of migrant populations, it is advisable to add extra protections when pursuing foreign approaches that focus on working conditions in parallel with international recruitment appear to have the most promise, such as Germany's 2019 introduction of the Care Wages Improvement Act and the Care Staff Strengthening Act (S87).

#### Supportive stage: outcomes that support worker performance

The supportive stage of the working lifespan was emphasized in the sources, in part due to the large number of outcomes in this category. Key outcomes in this stage include life-long learning, career advancement, working conditions, support systems, scope of practice, and compensation. Life-long learning was the most featured outcome in this set of interventions, with many evaluations of specific training programs or professional development workshops. Life-long learning can support job satisfaction (S116* and S117*), and care workers often have an interest in ongoing training, especially on topics such as dementia, and/or other specific conditions (S48*, S74*, and S141) and to learn delegated tasks (S55 and S146*).

Brief training workshops can successfully increase skills and knowledge of care workers (S12, S13*, S24, S27*, S55, S70*, S101, S103, S131, and S133*); some sources demonstrated that training is better delivered in a series and using a variety of modalities rather than a one-time workshop (S6*, S82, and S99*). There were some unique training approaches, such as adding new roles that also create career advancement opportunities. For example, establishing a knowledge broker role to translate research findings to practice across facilities (S9*) or creating a specialist dementia care worker role in institutional care settings who engages in a “quasi peer-to-peer approach” to expand the knowledge base of other workers (S74*).

Some sources highlighted the positive potential of career ladders tied to training opportunities and financial benefits (S1, S6, S17, S20*, S21*, S24, S33, S36*, S37*, S44*, S51, S68, S74*, S81, S85, S87, S110*, and S142*). Other sources stressed the importance of considering the demographic and socio-economic conditions of care workers to improve training completion and efficacy (S52, S54*, S78, and S82).

Some studies highlighted specific organizational philosophies and models associated with increased work satisfaction, for example, reablement (S59), health promotion (S81), and socially integrated day centers (S132*). Sources reporting information on supportive systems tended to focus on communication technologies with overall mixed results on whether technological interventions reduce work strain (S24, S38, S70*, S101, S128*, S131, and S133*) or enhance communication (S59, S60, S86, S89*, S93*, S94*, S96, S104*, S117*, S122, S128*, S129, and S136). Four studies, three of which were found in the gray literature, concluded that unionization and/or coalitions between workers and care recipients are effective mechanisms for improving working conditions, or recommended that governments should set labor standards aimed at the care sector (S46*, S87, S91, and S125*). Scheduling and security are important levers for intervening in working conditions in a variety of contexts (S43*, S46*, S59, S87, S110*, S142*, S146*, S147*, and S148). Varese and colleagues (S151*) highlight the value of mental health supports for both the care and health workforces.

Flexibility is important for the recruitment and retention of people who may be interested in care work if job conditions are accommodating to other commitments (S81, S141, and S142*). Flexibility in scheduling does not mean precarity. A Finnish study reports on health risks for care workers in precarious forms of employment (S68*), and a study out of the United States indicates that a stable work schedule is important for many workers (S110*). There is strong evidence that a positive organizational culture and supportive leadership can improve the conditions of work (S16*, S39*, S44*, S51, S59, S72*, S80*, S83, S84, S88*, S90, S98*, S110*, S114*, S117*, S124*, S126*, S130*, S132*, and S150). Outcomes categorized as “scope of practice” examined different models of skill mix and ongoing expansions of duties for care workers across varied settings (S2*, S7*, S17, S33, S38, S41, S50*, S51, S53, S59, S60, S74*, S75, S76, S83, S98*, S105, S107, S111, S117*, S119*, S121*, S122, S129*, and S146*). For example, 3 interventions conducted in the mid-2010s demonstrated the feasibility and value of training care workers in medication assistance (S2*, S75, and S118). The goal of many interventions was modifying workers' scope of practice, creating new roles or adjusting skill mix, demonstrating the adaptability of the health and care workforces (see also S9*, S12, S27, S32*, S36*, S39*, S45*, S48*, S52, S55, S71, S98*, S108, S115, S116*, and S149*).

There were no sources reporting on licensing care workers in the tradition of nursing or other health professions, and very few sources that reported on the regulation outcome. Partial mechanisms such as certification, registration, legal frameworks, and standardized educational requirements may be more effective (S14*, S71, S74, S85, S97, and S138).

The importance of wages and compensation is frequently mentioned in the literature review, discussion, and/or recommendation sections of the sources, but only appeared in 6.6% of the documented outcomes. Wage interventions that underwent evaluation demonstrated that better pay and consistency in wage structures can improve retention—that is, wages should be comparable regardless of employer type or work setting (S4*, S11*, S20*, S21*, S46*, S62, S87, S110*, S143*, S147*, and S150).

#### Exit stage: outcomes that support retention and pathways out of care work

There are 2 exit-stage-related outcomes—one focusing on retention, retirement, and succession planning, and the other on career choices and pathways out of care work. Most sources in this set reported on retention; 6 used training interventions as a mode of increasing retention (eg, S35* and S84) with limited success. For example, one residential facility in the United States implemented training to combat compassion fatigue. The training component worked as staff successfully gained knowledge about self-care strategies, but there were minimal improvements in retention over the study period, which was the underlying goal of the intervention (S40*). Expansive interventions (see below) appear to have the most promise for reducing turnover. For career choice, 2 sources reported on important policy innovations involving stepped-training pathways leading to careers in nursing or administration (S71 and S76). Multiple sources also recommended developing career advancement potential as an important lever to support retention and to increase integration between the health and care workforces (eg, S7*, S74*, and S141).

#### From entry to exit: interventions across the working lifespan

There were 18 sources reporting on highly complex interventions that targeted all stages of the working lifespan. For example, in 2013, an advocacy organization for direct care workers in New York piloted a multiyear intervention—the Homecare Aide Workforce Initiative—to improve skills, job satisfaction, and retention of home care workers (S44*). This multi-faceted intervention ranged from student selection and pre-service training to on-the-job support. Some innovative features include involving service providers in selecting students, using peer-instructors as teaching assistants, providing an enhanced curriculum that exceeded employer requirements, and stepped mentorship. The initiative improved retention, although the study emphasized that the training model was only one part of the solution, as improved wages and working conditions were also identified as key areas of impact. Four additional sources from Taiwan and the United States demonstrated that wage enhancement can support care workforces at all stages of working life (S21*, S110*, S142*, and S143*).

Merkel and colleagues (2019) looked at age-specific management strategies in 3 European contexts (S81). This cross-country analysis identified specific supportive practices, such as giving workers some control over their own schedules, and strategies aimed at retaining older workers. The findings suggest that age-specific strategies and flexible work options can improve workforce management and worker satisfaction.

Skills for Care is a “strategic workforce development and planning body” (S150, p. 8) in the United Kingdom that works across the public and private sectors, develops knowledge through comprehensive data collection, and delivers free resources for strategic and operational care workforce activities. The cross-sectoral planning body has had a growing impact on workforce issues since its inception in 2001, and when evaluated in 2024, this intervention addressed 9 thematic areas demonstrating a multi-pronged, cross-sectoral, and strategic approach to longstanding workforce issues.

#### Cross-cutting themes: addressing equity concerns

The additional outcomes include 2 categories, occupational segregation and ownership type. Care work remains largely segregated from other health and social professions in many contexts, and migration and racism can exacerbate inequities.^11^ There are limited interventions addressing this as an outcome. From the sources, transformative interventions account for socio-economic, global, racial, gendered, and other relevant factors in their design. For example, local-level educational interventions may help reduce workplace segregation by including English-language supports (S46* and S85) Scaling up, promising approaches include national legislation designed to protect migrant workers (S87), and providing supports for employers regarding inclusivity and ethical hiring of international workers (S150).

In terms of ownership, for-profit ownership appeared to have worse outcomes for workforce retention or was itself a barrier for implementing interventions (S21*, S29*, S35*, and S150). Policies that regulate service providers may reduce differences between public, nonprofit, and for-profit organizations (S20* and S69).

## Discussion

The sources we reviewed demonstrate a variety of interventions that successfully strengthen the care workforce. Training interventions are emphasized across both the entry and supportive working lifespan stages. The evidence on effective pedagogical approaches and formats is well-established.^5,19^ Based on the many examples of educational pilot studies, future efforts can focus on central coordination at national levels, adapted to country contexts. This aligns with other research that describes increasing educational accreditation for global health workforces, which can be used as a tool for “regulatory strengthening” in low- and middle-income countries and to support less formalized roles like care work.^4^ Further, many studies, especially in the life-long learning category, point out the importance of organizational leadership and contextual factors for ensuring the success of new programs, indicating there may be value in coordinating across organizations and emphasizing interprofessional training.

Many sources discuss working conditions and recommend better wages, and this is reflected in the broader literature on care work.^11,20^ Yet, only a few interventions were directed at increasing compensation for care workers across sectors (eg, S8*, S65*, S66, S134*, S142*, S143*), and most of these studies were conducted in the United States. Good jobs, regardless of the context, involve many factors, including stable hours, decent pay and consistency across work settings,^21^ balanced with flexibility—that is, options that allow people to work on their terms. Regardless of context, future interventions could pilot and evaluate a range of compensation models, including mixed approaches that combine stable salaries with performance-based incentives and non-monetary benefits such as career development opportunities, safe work environments, social protections, and work-life balance.

Needs identification, data, and evidence, occupational segregation, and professional regulation require further attention. Data and evidence are particularly key, as a strong knowledge base allows for monitoring, evaluation, and responsiveness in intervention planning. Regulation can have many meanings, including oversight from a professional college, educational accreditation, or legal frameworks governing a work sector. Other synthesis research on health workforce regulatory systems encourages cross-border recognition of qualifications and highlights “alternative models of occupational regulation” aimed at the unregulated workforce.^4^ This review underscores the importance of regulatory mechanisms, however, regulation of this sector in a manner similar to other health professions may not be beneficial to recruitment or growth (eg, S55, S141).

Other than cross-country comparisons, it is notable that there are only 3 sources in this review examining interventions in lower and lower-middle-income countries (LMICs)—Cameroon (S106*), India (S102) and Zimbabwe (S108)—and 2 sources from upper-middle-income countries—Brazil (S112*) and Thailand (S98*). LMICs may have younger populations and are focused on developing their primary health care workforces, but have a unique opportunity to design integrated workforces from the outset, avoiding some of the challenges faced by high-income countries that are trying to incorporate established care workforces within health systems. LMICs might face challenges in pursuing this dual development, such as resource constraints, political instability, or workforce shortages. There is a need for more research and reporting on cross-sectoral and cross-national interventions, especially considering the role of care workforce migration.

### Strengths, limitations, and future directions

The strength of this review lies in the use of the systematic realist methodology; however, our study was limited by the broad list of outcomes. While this approach allowed for a wide-ranging exploration of workforce issues, it led to a diluted focus on specific outcomes. Although the search was not geographically limited, the team only used English search terms, which may have overlooked relevant sources published in other languages. Efforts to translate sources partially mitigated this limitation.

In the screening stage, there were many sources with exceptional interventions that omitted care workers from the study or intervention design and thus were excluded from this review. We encourage researchers and policymakers to include care workers when designing clinical, workforce, or other interventions.

## Conclusion and recommendations

Based on the findings of this review, the WHO is carrying out a multi-stage and multi-country consultation and vetting process to develop global policy guidance on how to strengthen the care workforce and better integrate health and care workforce planning. The policy guidance will incorporate additional information from countries and regions that are underrepresented in the sources in this review.

At this juncture, the evidence gathered in this realist synthesis review indicates where policymakers can focus or begin care workforce interventions. Most notably, educational interventions are a core mechanism for recruitment and retention. Workforce planners are encouraged to focus on accreditation or oversight activities to help consolidate and systematize the field—for example, piloting core competencies, curriculum, or program standards tailored to local conditions yet aligning with global competency frameworks, or recognition of related training and experience.

The review also demonstrates the benefits of bundled interventions with mutually reinforcing components. Despite potential challenges, the most transformative and promising interventions looked at outcomes across all working lifespan stages or were associated with large system reforms that were evaluated iteratively. Not every element was successful, but these ambitious approaches were associated with compelling examples of effective interventions that helped to develop and support the care workforce. Returning to the example of educational oversight, interventions in this domain can result in outcomes that extend far beyond capacity building into recruitment, regulation, governance, and retention. Any evolution in educational requirements and professional development milestones could be aligned with a clear wage structure and perhaps a career ladder. Observing and accounting for the interdependent nature of key outcomes for the care workforce will enhance the success of any new intervention.

The evidence also supports policy interventions that experiment with varied wage models or alternative approaches to scheduling for care workers, recognizing how intervening in the material conditions of care will not only support workers, but will bolster the financial viability and reliability of services and ultimately population health outcomes. Policymakers should invest in pilot programs and longitudinal studies to explore innovative compensation strategies and their long-term effects on system costs, workforce stability, and the quality of care.

## Supplementary Material

qxaf128_Supplementary_Data
