# Supplementary material for: Interventions that strengthen the care workforce: a realist synthesis review
Source: Health Aff Sch. 2025 Jun 28;3(7):qxaf128. doi: 10.1093/haschl/qxaf128 (PMC12247801; doi:10.1093/haschl/qxaf128)
Supplement: qxaf128_Supplementary_Data [file qxaf128_supplementary_data.zip › Appendices.docx]

Appendices

Table of Contents

[A-1. Definition of care workers and developing the thematic areas of interest 2](#_Toc202205015)

[A-2. Search strings – academic literature 3](#_Toc202205016)

[A-3. Grey literature searches 7](#_Toc202205017)

[A-4. Inclusion and exclusion criteria 8](#_Toc202205018)

[A-5. Data extraction form 9](#_Toc202205019)

[A-6. PRISMA flow diagram 10](#_Toc202205020)

[A-7. Calculation and summary of QuADS scores 11](#_Toc202205021)

[A-8. Distribution of research methods in source material 12](#_Toc202205022)

[A-9. National context in source material 13](#_Toc202205023)

[A-10. Source list and full citations 14](#_Toc202205024)

[A-11. Source by thematic areas of interest based on the Working Lifespan framework 24](#_Toc202205025)

# Definition of care workers and developing the thematic areas of interest

**Definition of care workers:**

This review focuses on workers who provide direct and indirect care to people with needs across the life course due to disability, chronic illness, ageing, and/or acute health issues in formal or informal employment arrangements in public or private domains. There are a substantial number of people providing unpaid care, such as those tending to friends and family who are unwell, however, this review focuses on those providing care services to earn a living or maintain their livelihood, commonly referred to as paid care work (see figure below). The relevant four-digit ISCO (2008)(16) codes are 5321-Health care assistants; 5322-Home-based personal care workers; and 5152- domestic workers. (16)


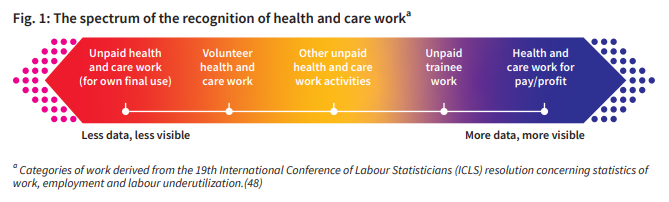


Source: Fair share for health and care: gender and the undervaluation of health and care work.

Geneva: World Health Organization; 2024. Licence: CC BY-NC-SA 3.0 IGO.

# Search strings – academic literature

1. **FINAL MEDLINE SEARCH**

Ovid MEDLINE(R) Epub Ahead of Print and In-Process, In-Data-Review & Other Non-Indexed Citations <March 12, 2024>

1 Health Workforce/ or workforce.ti. or social care.ti. or integrated.ti. 87697

2 home care services/ or home care services, hospital-based/ or homemaker services/ or Home health aides/ 39289

3 long-term care/ 28865

4 residential facilities/ or assisted living facilities/ or homes for the aged/ or nursing homes/ 51767

5 exp Household Work/ 4053

6 exp Nursing Assistants/ 4829

7 2 or 3 or 4 or 5 or 6 118355

8 (Care worker* or home care worker* or homecare worker* or home care aide* or homecare aide* or personal support worker* or personal care worker* or direct care worker* or domestic worker* or nurs* aide* or patient care assistant* or housekeeper* or healthcare aide* or health care aide* or health aide* or health care assistant* or healthcare assistant* or attendant* or direct care or integrated care work*).ti,ab,kf. 41493

9 7 or 8 156122

10 1 and 9 1645

11 exp Disabled Persons/ 74662

12 2 or 3 or 4 or 5 or 6 or 8 156122

13 11 and 12 and 1 30

14 (integrate or integrates or integrating or work* or organiz* or organis* or staff*).ti. 484980

15 social care.ti. 1905

16 14 and 15 295

17 (absenteeism or recruitment or retention or home care work* or care sector or care workers or direct care).ti. 67136

18 social care.ti,ab. 9237

19 17 and 18 117

20 10 or 13 or 16 or 19 1919

21 13 or 20 1919

22 (maternal or maternity or newborn* or neonat* or baby or babies or infant* or addict* or substance related or substance abuse* or substance use* or social work*).ti,ab,kf. 1264095

23 21 not 22 1772

24 (comment or editorial or news or newspaper article).pt. 1730028

25 (letter not (letter and randomized controlled trial)).pt. 1239698

26 24 or 25 2449591

27 23 not 26 1630

28 limit 27 to yr="2000 -Current" 1202

1. **FINAL EMBASE Search**

Embase <1974 to 2024 Week 10>

1 health workforce/ or workforce.ti. or social care.ti. or integrated.ti. 90097

2 exp home care/ 90770

3 long term care/ 150099

4 nursing home/ or residential home/ or assisted living facility/ or home for the aged/ 77831

5 (house work or household work*).ti,ab. 657

6 nursing assistant/ 5190

7 2 or 3 or 4 or 5 or 6 306230

8 (Care worker* or home care worker* or homecare worker* or home care aide* or homecare aide* or personal support worker* or personal care worker* or direct care worker* or domestic worker* or nurs* aide* or patient care assistant* or housekeeper* or healthcare aide* or health care aide* or health aide* or health care assistant* or healthcare assistant* or attendant* or direct care or integrated care work*).ti,ab,kf. 50731

9 7 or 8 352659

10 1 and 9 2245

11 exp disabled person/ 52382

12 2 or 3 or 4 or 5 or 6 or 8 352659

13 1 and 11 and 12 20

14 (integrate or integrates or integrating or work* or organiz* or organis* or staff*).ti. 514449

15 social care.ti. 2108

16 14 and 15 306

17 (absenteeism or recruitment or retention or home care work* or care sector or care workers or direct care).ti. 76274

18 social care.ti,ab. 11960

19 17 and 18 119

20 10 or 13 or 16 or 19 2535

21 13 or 20 2535

22 (maternal or maternity or newborn* or neonat* or baby or babies or infant* or addict* or substance related or substance abuse* or substance use* or social work*).ti,ab,kf. 1531240

23 21 not 22 2326

24 limit 23 to (article or article in press or "preprint (unpublished, non-peer reviewed)" or "review") 1666

25 limit 24 to yr="2000 -Current" 1501

1. **FINAL GLOBAL HEALTH Search**

Global Health <1973 to 2024 Week 11>

1 (workforce or social care or integrated).ti. 12330

2 home care/ or exp community health workers/ or home health aides/ 4739

3 long-term care/ 1430

4 nursing homes/ 4447

5 housework/ 73

6 2 or 3 or 4 or 5 10237

7 (Care worker* or home care worker* or homecare worker* or home care aide* or homecare aide* or personal support worker* or personal care worker* or direct care worker* or domestic worker* or nurs* aide* or patient care assistant* or housekeeper* or healthcare aide* or health care aide* or health aide* or health care assistant* or healthcare assistant* or attendant* or direct care or integrated care work*).ti,ab. 14728

8 6 or 7 24569

9 1 and 8 304

10 exp people with disabilities/ 8095

11 2 or 3 or 4 or 5 or 7 24569

12 1 and 10 and 11 3

13 (integrate or integrates or integrating or work* or organiz* or organis* or staff*).ti. 75028

14 social care.ti. 298

15 13 and 14 45

16 (absenteeism or recruitment or retention or home care work* or care sector or care workers or direct care).ti. 9060

17 social care.ti,ab. 1575

18 16 and 17 27

19 9 or 12 or 15 or 18 348

20 (maternal or maternity or newborn* or neonat* or baby or babies or infant* or addict* or substance related or substance abuse* or substance use* or social work*).ti,ab. 266380

21 19 not 20 286

22 limit 21 to journal article 279

23 limit 22 to yr="2000 -Current" 278

1. **FINAL CINAHL Search N=1.424**

S1 (MH "Workforce")

S2 TI (workforce OR "social care" or integrated)

S3 S1 OR S2

S4 TI ( “Care worker*” or “home care worker*” or “homecare worker*” or “home care aide*” or “homecare aide*” or “personal support worker*” or “personal care worker*” or “direct care worker*” or “domestic worker*” or “nurs* aide*” or “patient care assistant*” or housekeeper* or “healthcare aide*” or “health care aide*” or “health aide*” or “health care assistant*” or “healthcare assistant*” or attendant* or “direct care” or “integrated care work*” ) OR AB ( “Care worker*” or “home care worker*” or “homecare worker*” or “home care aide*” or “homecare aide*” or “personal support worker*” or “personal care worker*” or “direct care worker*” or “domestic worker*” or “nurs* aide*” or “patient care assistant*” or housekeeper* or “healthcare aide*” or “health care aide*” or “health aide*” or “health care assistant*” or “healthcare assistant*” or attendant* or “direct care” or “integrated care work*” ) OR SU ( “Care worker*” or “home care worker*” or “homecare worker*” or “home care aide*” or “homecare aide*” or “personal support worker*” or “personal care worker*” or “direct care worker*” or “domestic worker*” or “nurs* aide*” or “patient care assistant*” or housekeeper* or “healthcare aide*” or “health care aide*” or “health aide*” or “health care assistant*” or “healthcare assistant*” or attendant* or “direct care” or “integrated care work*” )

S5 S3 AND S4

S6 S3 AND S4

S7 TI ( (integrate or integrates or integrating or organiz* or organis* or staff*) ) AND TI "social care"

S8 TI “social care” OR AB “social care”

S9 TI (absenteeism or recruitment or retention or “home care work*” or “care sector” or “care workers” or “direct care”)

S10 S8 AND S9

S11 (MH “Home Health Care”) OR (MH “Home Visits”) OR (MH “Homemaker Services”) OR (MH “Respite Care”) OR (MH “Home Health Aides”) OR (MH “Long Term Care”) OR (MH “Residential Facilities”) OR (MH “Nursing Homes” ) OR (MH “Assisted Living”) OR (MH “Home Maintenance”) OR (MH “Certified Nursing Assistants”)

S12 S3 AND S11

S13 S3 AND S11

S14 TI ( maternal or maternity or newborn* or neonat* or baby or babies or infant* or addict* or ”substance related” or “substance abuse*” or “substance use*” or social work* ) OR AB ( maternal or maternity or newborn* or neonat* or baby or babies or infant* or addict* or ”substance related” or “substance abuse*” or “substance use*” or social work* )

S15 s13 NOT s14

S16 (MH “Persons with Disabilities”) OR (MH “Health Services for Persons with Disabilities”)

S17 (MH “Persons with Disabilities”) OR (MH “Health Services for Persons with Disabilities”)

S18 S5 OR S7 OR S10 OR S13

S19 S18 NOT S14

S20 S18 NOT S14

LIMITS APPLIED: 2000-2024; Academic Journals N=1424

# Grey literature searches

Google advanced search parameters:

1. All these words: see search words list
2. This exact word or phrase: blank
3. Any of these words: blank
4. None of these words: blank
5. Numbers ranging from: blank
6. Language: any language
7. Region: see region and VPN list
8. Last update: any time
9. Site: blank
10. Terms appearing: anywhere in the page
11. File type: adobe acrobat PDF or .ppt or .doc
12. Usage rights: not filtered by license

Search words list:

- - - 1. government (care-worker personal-care home-care homecare care-aide support-worker direct-care) workforce
      2. (non-profit not-for-profit NGO non-governmental) (care-worker home-care homecare care-aide support-worker domestic-worker patient-care-assistant health-aide health-care-assistant healthcare-assistant direct-care)

Region and VPN list:

1. None, none
2. India, India
3. Congo Brazzaville, Angola
4. Egypt, Egypt
5. Philippines, Philippines
6. Denmark, Denmark
7. USA, USA

# Inclusion and exclusion criteria

Inclusion criteria. Sources are eligible for inclusion if they:

1. Report empirical findings (qualitative, quantitative and/or mixed-methods results) related to a complex social intervention; AND​
2. Focus on one or more of the identified thematic areas of interest; AND​
3. Discuss care workers as defined above; AND ​
4. Are published after 1999 if it is a peer-reviewed publication OR are published after 2009 if it is a grey literature publication.

Exclusion criteria. Sources are excluded if they:

1. Do not report empirical findings related to a complex intervention (e.g., an opinion piece, a commentary, or summary of a study protocol without results); OR
2. Do not focus on one or more of the identified thematic areas of interest; OR
3. Focus on care workers who fall outside of the definition above; OR​
4. Focus on childcare for healthy children or services for people without direct care needs
5. Are published before the year 1999 if it is a peer-reviewed publication OR are published before 2009 if it is a grey literature publication.

# Data extraction form

1. Type of source
2. Research design
3. Research question or aims
4. Study population
5. Recruitment methods
6. Sample size/number of participants
7. Data collection methods and/or data source
8. Key findings and conclusions
9. Description of intervention
10. Country of intervention
11. Setting
12. Start year of study
13. Length of time of study
14. Start year of intervention
15. Length of time of intervention(s)
16. Contextual limitations
17. Describe theory or hypothesis
18. Is the theory of change explicit or inferred?
19. Outcome types
20. Entry Outcome categories
21. Data and evidence
    1. Needs identification
    2. Needs identification
    3. Funding
    4. Pre-service education requirements
    5. Recruitment
22. Supportive Outcome categories
    1. Professional regulation
    2. Organizational leadership
    3. Working conditions
    4. Scope of practice
    5. Supports systems
    6. Compensation/remuneration and benefits
    7. Life-long learning
    8. Career advancement
    9. Community integration
23. Exit Outcome categories
    1. Retirement/succession planning
    2. Career choice/Career pathways out of care work
24. Additional/cross-cutting equity outcomes
25. Occupational segregation
26. Public vs Private employment
27. Other

#
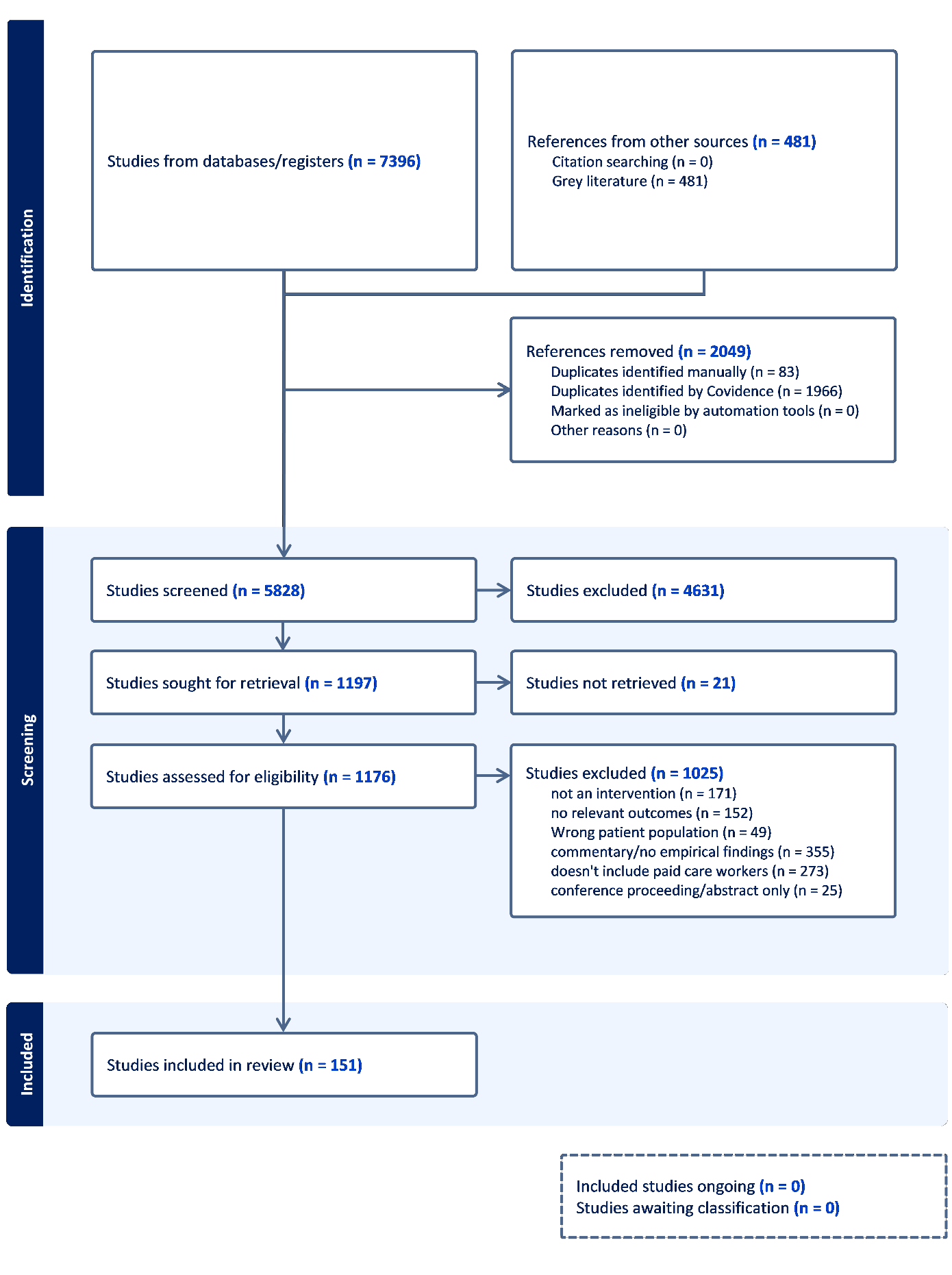
 PRISMA flow diagram

# Calculation and summary of QuADS scores

The QuADS tool (Quality Assessment for Diverse Studies) was developed to assess multi-method research. QuADS scores are calculated using a rubric of 13 quality criteria, each marked on a scale of 0 to 3 then summed to reach the quality score. The lowest possible score is thus 0 and the highest possible is 39. The authors caution against setting a given score as low or high quality as “any cut-off would be arbitrary and not appropriate when using this tool”. As such, we report study quality in terms of its quartile score within the sample.

Source reference: 1. Harrison R, Jones B, Gardner P, Lawton R. Quality assessment with diverse studies (QuADS): an appraisal tool for methodological and reporting quality in systematic reviews of mixed- or multi-method studies. *BMC Health Serv Res*. 2021;21(1):1-20. doi:10.1186/s12913-021-06122-y

All studies were scored independently by two researchers, and any discrepancies were discussed by the team for consensus or resolved by a third researcher.

As shown in the chart below, the studies included in this review are generally of high quality with a median score of 30 out of 39 and only 17 studies scoring less than 20.


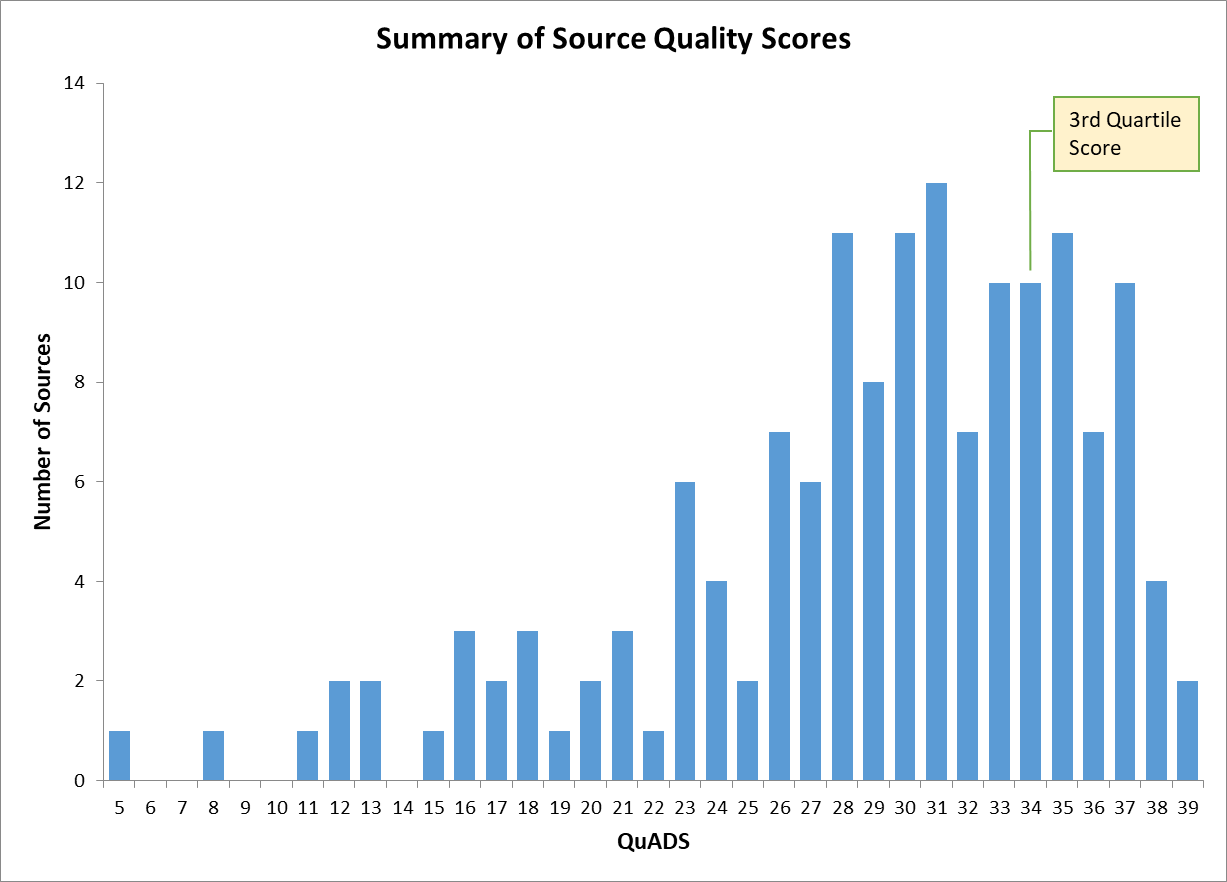


# Distribution of research methods in source material

“Other” methods refers to ethnographies, policy analyses, case studies, and case comparisons.

# National context in source material

| **National context of the intervention** | **# sources** | **Remarks** | | |
| --- | --- | --- | --- | --- |
| USA | 46 | The USA and the UK dominate as the national context in the source material | 17 countries serve as the context for the bulk of the source material | |
| UK | 38 |  |  |  |
| Australia | 18 |  |  |  |
| Canada | 15 |  |  |  |
| Netherlands | 13 |  |  |  |
| Norway | 7 |  |  |  |
| Sweden | 6 |  |  |  |
| Finland | 4 |  |  |  |
| Denmark, France, Germany, Taiwan | 3 |  |  |  |
| India, Ireland, Japan, Poland, Spain | 2 |  |  |  |
| Austria, Belgium, Brazil, Bulgaria, Cameroon, Chile, Columbia, Croatia, Cyprus, Czech Republic, Estonia, Fiji, Greece, Hungary, Italy, Latvia, Lithuania, Luxembourg, Malta, New Zealand, Portugal, Romania, South Korea, Slovakia, Slovenia, South Africa, Switzerland, Thailand, Zimbabwe | 1 | Brazil, Cameroon, New Zealand, South Korea, Switzerland, Thailand and Zimbabwe served as the sole context of a given source. The remainder were multi-country comparisons. 18 of these countries were mentioned in a single source (Kroezen2018). | | |
| Note: 10 sources involved international comparisons of 2 or more countries | | | |  |

# Source list and full citations

| **Source ID** | **Source** | **Citation** |
| --- | --- | --- |
| S1 | Aldeghi 2013 | Aldeghi I, Galdemar V, Gilles L. More and better jobs in home-care services: France [Internet]. Dublin: Eurofound; 2013 Sep [cited 2024 Jul 9] p. 50. Available from: <https://www.eurofound.europa.eu/en/publications/2013/more-and-better-jobs-home-care-services> |
| S2 | Arain 2017 | Arain MA, Deutschlander S, Charland P. Are healthcare aides underused in long-term care? A cross-sectional study on continuing care facilities in Canada. BMJ Open. 2017;7(5):e015521. |
| S3 | Ashwood 2016 | Ashwood L, Hughes J, Macrae A, Marsden P. Using a nurse leads team to spearhead workforce change. Practice Nursing. 2016 Apr 2;27(4):194–6. |
| S4 | Australia 2021 | Australian Government. Care workforce labour market study [Internet]. Canberra: Australian Government; 2021 Sep [cited 2024 Jul 8] p. 475. Available from: <https://www.jobsandskills.gov.au/sites/default/files/2023-12/Care%20Workforce%20Labour%20Market%20Study_0.pdf> |
| S5 | Bandini 2024 | Bandini JI, Rollison J, Etchegaray J. Understanding multilevel factors related to retention among the direct care workforce: Incorporating lessons learned in considering innovative interventions. J Healthc Manag. 2024;69(1):59–73. |
| S6 | Barnett 2018 | Barnett K, Moretti C, Howard S. Enabling aged care teaching and research: The TRACS footprint. Journal of Research in Nursing. 2018;23(2/3):267–87. |
| S7 | Basnight 2023 | Basnight R, Berry P, Capes K, Pearce S, Thompson J, Allen DH, et al. Evaluation of lay health workers on quality of care in the inpatient setting. PloS One. 2023;18(11):e0293068. |
| S8 | Baughman 2010 | Baughman RA, Smith K. The effect of Medicaid wage pass-through programs on the wages of direct care workers. Med Care. 2010;48(5):426–32. |
| S9 | Bayly 2018 | Bayly M, Forbes D, Blake C, Peacock S, Morgan D. Development and implementation of dementia-related integrated knowledge translation strategies in rural home care. Online Journal of Rural Nursing & Health Care. 2018;18(2):29–64. |
| S10 | Bernard 2005 | Bernard S. A national survey of adult placement schemes in England: Recruitment and retention of adult placement carers. Health Soc Care Comm. 2005;13(6):563–9. |
| S11 | Bjerregaard 2015 | Bjerregaard K, Haslam SA, Morton T, Ryan MK. Social and relational identification as determinants of care workers’ motivation and well-being. Front Psychol. 2015;6(101550902):1460. |
| S12 | Boscart 2018 | Boscart VM, Heckman G, Davey M, Heyer M, Hirdes JP. Impact of the applied simulated and integrated learning approach on nursing assistants’ knowledge and confidence caring for frail seniors in nursing homes. Pilot and Feasibility Studies. 2018;4(101676536):77. |
| S13 | Boscart 2019 | Boscart VM, Crutchlow L, Taucar LS, Schelling S, Fung E, Betini RSD, et al. Improving clinical care outcomes for Canadian seniors: Findings of a pilot study evaluating an applied simulated and integrated learning approach (ASILA) for home care workers. Educ Gerontol. 2019;45(10):612–23. |
| S14 | Boult 2011 | Boult C, Reider L, Leff B, KD F, CM B, JL W, et al. The effect of guided care teams on the use of health services: Results from a cluster-randomized controlled trial. Arch Intern Med. 2011;171(5):460–6. |
| S15 | Boumans 2008 | Boumans NPG, Berkhout AJMB, Vijgen SMC, Nijhuis FJJN, Vasse RM. The effects of integrated care on quality of work in nursing homes: A quasi-experiment. Int J Nurs Stud. 2008;45(8):1122–36. |
| S16 | Brown 2016 | Brown PB, Hudak SL, Horn SD, Cohen LW, Reed DA, Zimmerman S. Workforce characteristics, perceptions, stress, and satisfaction among staff in Green House and other nursing homes. Health Serv Res. 2016;51(S1):418–32. |
| S17 | Bunn 2020 | Bunn C, Harwood E, Akhter K, Simmons D. Integrating care: the work of diabetes care technicians in an integrated care initiative. BMC Health Serv Res. 2020;20(1):1–11. |
| S18 | Burgdorf 2020 | Burgdorf J, Wolff J, Willink A, Woodcock C, Davis K, Stockwell I. Expanding Medicaid coverage for community-based long-term services and supports: Lessons from Maryland's Community First Choice program. J Appl Gerontol. 2020;39(7):745–50. |
| S19 | Chapman 2023 | Chapman SA, Miller JR, Spetz J. Emerging Health Technologies in Long-Term Care and Suppliers’ Views on Their Potential to Assist and Support the Workforce. Med Care Res Rev. 2023;80(6):619–30. |
| S20 | Charlsworth 2024 | Charlesworth S, Cunningham I, Daly T. Decent work and quality long-term care systems [Internet]. Public Services International (PSI); 2024 Jan [cited 2024 Jul 9] p. 77. Available from: <https://publicservices.international/resources/digital-publication/decent-work-and-quality--br-long-term-care-systems-br-full-report?id=14383&lang=en> |
| S21 | Chen 2014 | Chen C.-F., Fu T.-H. Policies and transformation of long-term care system in Taiwan. Ann Geriatr Med Res. 2020;24(3):187–94. |
| S22 | Chen 2020 | Chen HL. Care workers in long-term care for older people: challenges of quantity and quality. Eur J Soc Work. 2014;17(3):383–401. |
| S23 | Chester 2014 | Chester H, Hughes J, Challis D. Commissioning social care for older people: influencing the quality of direct care. Ageing Soc. 2014;34(6):930–50. |
| S24 | Choy 2016 | Choy S, Henderson A. Preferred strategies for workforce development: feedback from aged care workers. Aust Health Rev. 2016;40(5):533–7. |
| S25 | Christensen 2017 | Christensen K, Hussein S, Ismail M. Migrants’ decision-process shaping work destination choice: the case of long-term care work in the United Kingdom and Norway. Eur J Ageing. 2017;14(3):219–32. |
| S26 | Clapper 2023 | Clapper Y, ten Hove W, Bekker R, Moeke D. Team size and composition in home healthcare: Quantitative insights and six model-based principles. Healthcare. 2023;11(22):2935. |
| S27 | Clarke 2019 | Clarke S, Wood L, Farrell A, Evison P, Stupple P. Developing a course for primary care healthcare assistants in recognising and responding to deteriorating patients. Primary Health Care. 2019;29(2):32–6. |
| S28 | Coogle 2007 | Coogle CL, Parham IA, Jablonski R, Rachel JA. Enhanced care assistant training to address the workforce crisis in home care: changes related to job satisfaction and career commitment. Care Management Journals. 2007;8(2):71–81. |
| S29 | Cook 2017 | Cook G, McNall A, Thompson J, Hodgson P, Shaw L, Cowie D. Integrated working for enhanced health care in English nursing homes. J Nurs Scholarship. 2017;49(1):15–23. |
| S30 | Craswell 2020 | Craswell A, Wallis M, Coates K, Marsden E, Taylor A, Broadbent M, et al. Enhanced primary care provided by a nurse practitioner candidate to aged care facility residents: A mixed methods study. Collegian. 2020;27(3):281–7. |
| S31 | DeGraves 2024 | DeGraves BS, Titley H, Duan Y, Thorne TE, Banerjee S, Ginsburg L, et al. Workforce resilience supporting staff in managing stress: A coherent breathing intervention for the long-term care workforce. J Am Geriatr Soc. 2024;72(3):753–66. |
| S32 | Denton 2015 | Denton M, Brookman C, Zeytinoglu I, Plenderleith J, Barken R. Task shifting in the provision of home and social care in Ontario, Canada: Implications for quality of care. Health Soc Care Comm. 2015;23(5):485–92. |
| S33 | Deshong 2010 | Deshong D, Henderson A. The trainee assistant in nursing: A pilot exercise in building and retaining a workforce. Aust Health Rev. 2010;34(1):41–3. |
| S34 | Devine 2006 | Devine M, Cuming P, Magennis S, Beattie M, Moore G, Fitzsimmons M. Actioning health in Struell Lodge for people with learning disabilities. Foundation of Nursing Studies. 2006;3(4):1–4. |
| S35 | Dill 2010 | Dill JS, Morgan JC, Konrad TR. Strengthening the long-term care workforce: The influence of the Win a Step Up workplace intervention on the turnover of direct care workers. J Appl Gerontol. 2010;29(2):196–214. |
| S36 | Dill 2014 | Dill JS, Chuang E, Morgan JC. Healthcare organization-education partnerships and career ladder programs for health care workers. Soc Sci Med. 2014;122(ut9, 8303205):63–71. |
| S37 | Dill 2022 | Dill JS, Morgan JC, Van Heuvelen J, Gingold M. Professional certification and earnings of health care workers in low social closure occupations. Soc Sci Med. 2022;303(1):115000. |
| S38 | Dix 2023 | Dix S, Rawson H, Russo P, Team V, Griffiths D, Morphet J. Practical infection control training for Victoria’s aged care workforce at the time of COVID-19 pandemic: a community case study. Front Public Health. 2023;11(101616579):1155980. |
| S39 | Douglas 2023 | Douglas NF, Browning S, Claypool K. Preliminary evidence for dementia collaborative coaching. Am J Speech Lang Pathol. 2023;32(5):2146–57. |
| S40 | Dreher 2019 | Dreher MM, Hughes RG, Handley PA, Tavakoli AS. Improving Retention Among Certified Nursing Assistants Through Compassion Fatigue Awareness and Self-Care Skills Education. J Holist Nurs. 2019;37(3):296–308. |
| S41 | Dryden 2009 | Dryden H, Addicott R. Evaluation of a pilot study day for healthcare assistants and social care officers. Int J Palliat Nurs. 2009;15(1):6–11. |
| S42 | Edes 2014 | Edes T, Kinosian B, Vuckovic NH, Olivia Nichols L, Mary Becker M, Hossain M. Better access, quality, and cost for clinically complex veterans with home-based primary care. J Am Geriatr Soc. 2014;62(10):1954–61. |
| S43 | Elbourne 2015 | Elbourne HF, le May A. Crafting intermediate care: one team’s journey towards integration and innovation. J Res Nurs. 2015;20(1):56–71. |
| S44 | Feldman 2019 | Feldman PH, Ryvicker M, Evans LM, Barrón Y. The homecare aide workforce initiative: Implementation and outcomes. J Appl Gerontol. 2019;38(2):253–76. |
| S45 | Finnema 2005 | Finnema E, Dröes RM, Ettema T, Ooms M, Adèr H, Ribbe M, et al. The effect of integrated emotion-oriented care versus usual care on elderly persons with dementia in the nursing home and on nursing assistants: A randomized clinical trial. Int J Geriatr Psych. 2005;20(4):330–43. |
| S46 | Fischer 2020 | Fischer SH, McBain RK, Faherty LJ, Sousa JL, Kareddy V, Gittens AD, et al. Strengthening the entry-level health care workforce: Finding a path [Internet]. Washington, DC: RAND Health Care; 2020 Sep [cited 2024 Jul 9] p. 242. Available from: <https://aspe.hhs.gov/reports/strengthening-entry-level-health-care-work-force-finding-path> |
| S47 | Florek 2022 | Florek K. Registration of health care assistants: An investigation of benefits and drawbacks  based on EPSU affiliates’ experiences [Internet]. Brussles, Belgium: European Public Service Union; 2022 Oct [cited 2024 Jul 9] p. 28. Available from: <https://www.epsu.org/sites/default/files/article/files/Registration%20of%20Health%20Care%20Assistants_b.pdf> |
| S48 | Fong 2022 | Fong MC, Russell D, Brickner C, Gao O, Vito S, McDonald M. Medicaid long-term care workforce training intervention and value-based payment metrics. Health Serv Res. 2022;57(2):340–50. |
| S49 | Franzosa 2023 | Franzosa E, Judon KM, Gottesman EM, Koufacos NS, Runels T, Augustine M, et al. Improving care coordination between veterans health administration primary care teams and community home health aide providers: A qualitative study. J Appl Gerontol. 2023;42(4):552–60. |
| S50 | Gaber 2020 | Gaber J, Oliver D, Valaitis R, Cleghorn L, Lamarche L, Avilla E, et al. Experiences of integrating community volunteers as extensions of the primary care team to help support older adults at home: A qualitative study. BMC Fam Pract. 2020;21(1):1–13. |
| S51 | Gleason 2024 | Gleason LJ, Long M, Graupner J, Kroplewski R, Gower P, Mittal K, et al. Development and implementation of a novel skilled nursing facility certified nursing assistant leadership academy. J Am Geriatr Soc. 2024;72(1):323–5. |
| S52 | Guerrero 2020 | Guerrero LR, Richter Lagha R, Shim A, Gans D, Schickedanz H, Shiner L, et al. Geriatric workforce development for the underserved: Using RCQI methodology to evaluate the training of IHSS caregivers. J Appl Gerontol. 2020;39(7):770–7. |
| S53 | Håland 2021 | Håland E, Røsstad T, Osmundsen TC. Care pathways as boundary objects between primary and secondary care: Experiences from Norwegian home care services. Health. 2015;19(6):635–51. |
| S54 | Hald 2021 | Hald A.N., Bech M., Burau V. Conditions for successful interprofessional collaboration in integrated care - Lessons from a primary care setting in Denmark. Health Pol. 2021;125(4):474–81. |
| S55 | Hamer 2018 | Hamer F, Ponto M. Educating the workforce: improving the education and competencies of healthcare assistants in an acute surgical unit. Brit J Healthcare Assistants. 2018;12(1):42–7. |
| S56 | Hanlon 2007 | Hanlon N, Rosenberg M, Clasby R. Offloading social care responsibilities: recent experiences of local voluntary organisations in a remote urban centre in British Columbia, Canada. Health Soc Care Comm. 2007;15(4):343–51. |
| S57 | Hanssen 2017 | Hanssen H, Norheim A, Hanson E. How can web-based training facilitate a more carer friendly practice in community-based health and social care services in Norway? Staff experiences and implementation challenges. Health Soc Care Comm. 2017;25(2):559–68. |
| S58 | Harlock 2020 | Harlock J, Caiels J, Marczak J, Peters M, Fitzpatrick R, Wistow G, et al. Challenges in integrating health and social care: the Better Care Fund in England. J Health Serv Res Policy. 2020;25(2):86–93. |
| S59 | Hjelle 2016 | Hjelle KM, Skutle O, Førland O, Alvsvåg H. The reablement team’s voice: A qualitative study of how an integrated multidisciplinary team experiences participation in reablement. J Multidiscipl Health. 2016;9(1):575–85. |
| S60 | Hjelm 2000 | Hjelm K, Nyberg P, Apelqvist J. Chronic leg ulcers in Sweden: A survey of wound management. J Wound Care. 2000;9(3):131–6. |
| S61 | Hollinger-Smith 2001 | Hollinger-Smith L, Ortigara A, Lindeman D. Developing a comprehensive long-term care workforce initiative. Alzheimer’s Care Quarterly. 2001;2(3):33–40. |
| S62 | Huang 2020 | Huang SS, Bowblis JR. Workforce retention and wages in nursing homes: An analysis of managerial ownership. J Appl Gerontol. 2020;39(8):902–7. |
| S63 | Hult 2023 | Hult M, Kallio H, Kangasniemi M, Pesonen T, Kopra J. The effects of precarious employment and calling on the psychosocial health and work well-being of young and older workers in the care sector: a longitudinal study. Int Arch Occ Env Hea. 2023;96(10):1383–92. |
| S64 | Kemper 2008 | Kemper P, Brannon D, Barry T, Stott A, Heier B. Implementation of the Better Jobs Better Care demonstration: Lessons for long-term care workforce initiatives. Gerontologist. 2008;48(suppl-1):26–35. |
| S65 | Khavjou 2024 | Khavjou O, Suarez G, Tyler D, Squillace M, Dey J, Oliveira I. Direct care workforce experienced limited wage improvements despite state policy efforts [Internet]. Washington, DC: Office of the Assistant Secretary for Planning and Evaluation, US Department of Health and Human Services; 2024 Jan [cited 2024 Jul 18] p. 12. Available from: <https://aspe.hhs.gov/sites/default/files/documents/328622aa1a80819273a80a16d196a3ba/state-efforts-improve-dcw-wages-ib.pdf> |
| S66 | Kim 2019 | Kim J. Informal employment and the earnings of home-based home care workers [Internet]. Pascataway, New Jersey: Rutgers School of Management and Labor Relations; 2019 [cited 2024 Jul 5] p. 36. Available from: <https://smlr.rutgers.edu/sites/default/files/Documents/Centers/CWW/Publications/informal_employment_and_the_earnings_of_home-based_home_care_workers.pdf> |
| S67 | Kornas 2021 | Kornas K, O’Neill M, Liang CY, Diemert L, Ayanian T, Chang M, et al. Health care providers’ experiences with delivering person centred care in an integrated care program for thoracic surgery patients in Ontario, Canada. J Integr Care. 2021;29(3):346–56. |
| S68 | Kroezen 2018 | Kroezen M, Schafer W, Sermeus W, Hansen J, Batenburg R. Healthcare assistants in EU member states: An overview. Health Pol. 2018;122(10):1109–17. |
| S69 | Kubo 2014 | Kubo M. Long-term care insurance and market for aged care in Japan: focusing on the status of care service providers by locality and organisational nature based on survey results. Australas J Ageing. 2014;33(3):153–7. |
| S70 | Kulnik 2017 | Kulnik ST, Pöstges H, Brimicombe L, Hammond J, Jones F. Implementing an interprofessional model of self-management support across a community workforce: A mixed-methods evaluation study. J Interprof Care 2017;31(1):75–84. |
| S71 | Lacher 2015 | Lacher S, Geest S, Denhaerynck K, Trede I, Ausserhofer D. The quality of nurses' work environment and workforce outcomes from the perspective of Swiss allied healthcare assistants and registered nurses: A cross-sectional survey. J Nurs Scholarship 2015;47(5):458–67. |
| S72 | Larsson 2014 | Larsson R, Ljungblad C, Sandmark H, Åkerlind I. Workplace health promotion and employee health in Swedish municipal social care organizations. J Public Health. 2014;22(3):235–44. |
| S73 | Lawlis 2016 | Lawlis T, Wicks A, Jamieson M, Haughey A, Grealish L. Interprofessional education in practice: Evaluation of a work integrated aged care program. Nurse Educ Pract. 2016;17:161–6. |
| S74 | Lea 2023 | Lea EJ, Robinson AL, Doherty KV. From residential aged care worker to dementia care support worker: A qualitative study of senior aged care staff perceptions of the role. Aust J Adv Nurs. 2023;40(4):13–21. |
| S75 | Lee 2015 | Lee CY, Beanland C, Goeman D, Johnson A, Thorn J, Koch S, et al. Evaluation of a support worker role, within a nurse delegation and supervision model, for provision of medicines support for older people living at home: the Workforce Innovation for Safe and Effective (WISE) Medicines Care study. BMC Health Serv Res. 2015;15(1):1–11. |
| S76 | Lokmic-Tomkins 2021 | Lokmic-Tomkins Z, Khor MKY, Matthews KA, Martin JA, McGillion A. Improving the health assistant in nursing employment model through entry to practice nursing student perceptions: a cross-sectional study. Contemp Nurse. 2021;57(6):472–81. |
| S77 | LTSS 2018 | LTSS Centre. Filling the care gap: Integrating foreign-born nurses and personal care assistants into the field of long-term services and supports [Internet]. Washington: The Global Ageing Network & Leading Age LTSS Centre @UMass Boston; 2018 [cited 2024 Jul 9] p. 36. Available from: <https://leadingage.org/wp-content/uploads/drupal/LA_SodexoReport2018_Digital_r2.pdf> |
| S78 | Luz 2015 | Luz C, Hanson K. Training the personal and home care aide workforce: Challenges and solutions.. Home Health Care Manag Pract. 2015;27(3):150–3. |
| S79 | Manheim 2021 | Manheim C, Haverhals L, Gilman C, Karuza J, Olsan T, Edwards S, et al. VA home based primary care teams: Partnering with and acting as caregivers for veterans. Home Health Care Serv Q. 2021;40(1):1–15. |
| S80 | McDaniel 2011 | McDaniel C, Roche JK, Veledar E. Ethics environment in long-term care. J Appl Gerontol. 2011;30(1):67–84. |
| S81 | Merkel 2019 | Merkel S, Ruokolainen M, Holman D. Challenges and practices in promoting (ageing) employees working career in the health care sector - case studies from Germany, Finland and the UK. BMC Health Serv Res. 2019;19:1-2. |
| S82 | Meyer 2018 | Meyer C, McMillan S, Browning C, Appannah A, Ogrin R. Design of an evidence-based diversity workshop to support participation of older people in their community care. Educ Gerontol. 2018;44(5/6):391–402. |
| S83 | Monro 2021 | Monro C, Mackenzie L, O’Loughlin K, Low L. Perspectives of operational staff working in residential care and aged care reforms. Nurs Health Sci. 2021;23(4):948–56. |
| S84 | Morgan 2008 | Morgan JC, Edris N, Luz CC, Ochylski DP, Stineman A, Winchester L, et al. Testing U.S. state-based training models to meet health workforce needs in long-term care. Ageing International. 2018;43(1):123–40. |
| S85 | Morgan 2018 | Morgan JC, Konrad TR. A mixed-method evaluation of a workforce development intervention for nursing assistants in nursing homes: The case of Win a Step Up. Gerontologist. 2008;48 Spec No 1(fp5, 0375327):71–9. |
| S86 | Mun 2023 | Mun H, Cho K, Lee S, Choi Y, Oh SJ, Kim YS, et al. Patient-centered integrated model of home health care services in South Korea (PICS-K). Int J Integr Care. 2023;23(2):1–11. |
| S87 | Murphy 2022 | Murphy L, Farragher L, Long J. The role, function, and supply of home care workers in four European countries [Internet]. Dublin: Health Research Board; 2022 Aug [cited 2024 Jul 7]. Available from: <https://www.gov.ie/en/publication/5412b-evidence-brief-the-role-function-and-supply-of-home-care-workers-in-four-european-countries/> |
| S88 | Naccarella 2018 | Naccarella L, Newton C, Pert A, Seemann K, Williams R, Sellick K, et al. Workplace design for the Australian residential aged care workforce. Australas J Ageing. 2018;37(3):194–201. |
| S89 | Nadav 2021 | Nadav J, Kaihlanen AM, Kujala S, Laukka E, Hilama P, Koivisto J, et al. How to implement digital services in a way that they integrate into routine work: Qualitative interview study among health and social care professionals. J Med Internet Res. 2021;23(12):N.PAG-N.PAG. |
| S90 | Nandram 2014 | Nandram S, Koster N. Organizational innovation and integrated care: lessons from Buurtzorg. J Integr Care. 2014;22(4):174–84. |
| S91 | Navarra 2023 | Navarra C, Saraceno C, Gromada A, Bell T, Marzo C, Lebrun JF, et al. The European care strategy: A chance to ensure inclusive care for all? [Internet]. Brussels: The Foundation for European Progressive Studies & the Friedrich-Ebert Stifung; 2023 Mar [cited 2024 Jul 9] p. 140. Available from: <https://feps-europe.eu/publication/the-european-care-strategy/> |
| S92 | Norman 2018 | Norman GJ, Orton K, Wade A, Morris AM, Slaboda JC. Operation and challenges of home-based medical practices in the US: Findings from six aggregated case studies. BMC Health Serv Res. 2018;18:1-N.PAG. |
| S93 | Obayashi 2020 | Obayashi K, Masuyama S. Pilot and feasibility study on elderly support services using communicative robots and monitoring sensors integrated with cloud robotics.. BMC Health Serv Res. 2020;42(2):364-371.e4. |
| S94 | Ochieng 2022 | Ochieng L, Salehi M, Ochieng R, Nijhof D, Wong R, Gupta V, et al. Augmented video consultations in care homes during the COVID-19 pandemic: A qualitative study. BJGP Open. 2022;6(4):1–9. |
| S95 | Ochylski 2017 | Ochylski DP, Luz CC, Shen X. Direct care workforce training: Internet accessibility and acceptance. J Gerontol Nurs. 2017;43(6):46–52. |
| S96 | Ornstein 2011 | Ornstein K, Smith KL, Foer DH, Lopez-Cantor MT, Soriano T. To the hospital and back home again: a nurse practitioner-based transitional care program for hospitalized homebound people. J Am Geriatr Soc. 2011;59(3):544–51. |
| S97 | Øvretveit 2010 | Øvretveit J, Hansson J, Brommels M. An integrated health and social care organisation in Sweden: Creation and structure of a unique local public health and social care system. Health Policy. 2010;97(2/3):113–21. |
| S98 | Pagaiya 2021 | Pagaiya N, Noree T, Hongthong P, Gongkulawat K, Padungson P, Setheetham D. From village health volunteers to paid care givers: the optimal mix for a multidisciplinary home health care workforce in rural Thailand. Hum Resour Health. 2021;19(1):1–10. |
| S99 | Parveen 2021 | Parveen S, Smith SJ, Sass C, Oyebode JR, Capstick A, Dennison A, et al. Impact of dementia education and training on health and social care staff knowledge, attitudes and confidence: a cross-sectional study. BMJ Open. 2021;11(1):e039939. |
| S100 | Paulus 2005 | Paulus ATG, van Raak A, Keijzer F. Informal and formal caregivers’ involvement in nursing home care activities: impact of integrated care. J Adv Nurs. 2005;49(4):354–66. |
| S101 | Pointu 2005 | Pointu A, Cole C. An education programme for social care staff: improving the health of people who have a learning disability and epilepsy. Brit J Learn Disabil. 2005;33(1):39–43. |
| S102 | Poulain 2023 | Poulain M, Herm A, Cantisani G. Elderly caregiving sector: India-Europe labour migration [Internet]. Geneva: International Organization for Migration; 2023 Jan [cited 2024 Jun 18] p. 46. Available from: <https://india.iom.int/sites/g/files/tmzbdl2296/files/documents/2024-06/elderly-care-report.pdf> |
| S103 | Reymond 2005 | Reymond L, Charles M, Israel F, Read T, Treston P. A strategy to increase the palliative care capacity of rural primary health care providers. Aust J Rural Health. 2005;13(3):156–61. |
| S104 | Robben 2012 | Robben SH, Perry M, Huisjes M, van Nieuwenhuijzen L, Schers HJ, van Weel C, et al. Implementation of an innovative web-based conference table for community-dwelling frail older people, their informal caregivers and professionals: a process evaluation. BMC Health Serv Res. 2012;12(1):251–251. |
| S105 | Robertson 2023 | Robertson S, King R, Taylor B, Laker S, Wood E, Senek M, et al. Primary care trainee nursing associates in England: A qualitative study of higher education institution perspectives. Prim Health Care Res. 2023;24:1–8. |
| S106 | Robyn 2015 | Robyn PJ, Shroff Z, Zang OR, Kingue S, Djienouassi S, Kouontchou C, et al. Addressing health workforce distribution concerns: a discrete choice experiment to develop rural retention strategies in Cameroon. Int J Health Policy Manag. 2015;4(3):169–80. |
| S107 | Rodgers 2017 | Rodgers V, Marshall B, Hey F, Blackwell A, Lewer P. Readiness for providing primary palliative care in regional aged residential care: Partnering with SEQUAL specialist palliative care nurses. Nurs Prax N Z. 2017;33(3):31–40. |
| S108 | Rödlach 2009 | Rödlach A. Home-based care for people living with AIDS in Zimbabwe: voluntary caregivers’ motivations and concerns. Afr J AIDS Res. 2009;8(4):423–31. |
| S109 | Røsstad 2017 | Røsstad T, Salvesen Ø, Steinsbekk A, Grimsmo A, Sletvold O, Garåsen H. Generic care pathway for elderly patients in need of home care services after discharge from hospital: A cluster randomised controlled trial. BMC Health Serv Res. 2017;17:1–9. |
| S110 | Russell 2022 | Russell D, Fong MC, Gao O, Lowenstein D, Haas M, Wiggins F, et al. Formative evaluation of a workforce investment organization to provide scaled training for home health aides serving managed long-term care plan clients in New York state. J Appl Gerontol. 2022;41(7):1710–21. |
| S111 | Sandoz 2019 | Sandoz H, Warner J, Ovens E. Transformative education to improve wound care and sustain workforce. Wounds UK. 2019;15(4):48–53. |
| S112 | Savassi 2021 | Savassi L.C.M., Dias M.B., Boing A.F., Verdi M., Lemos A.F. Educational strategies for human resources in home health care: 8 years’ experience from Brazil. Revista Panamericana de Salud Pública. 2020;44 |
| S113 | Schoville 2020 | Schoville R, Titler MG. Integrated technology implementation model: Examination and enhancements. Comput Inform Nurs. 2020;38(11):579–89. |
| S114 | Sexton 2021 | Sexton JB, Adair KC, Profit J, Bae J, Rehder KJ, Gosselin T, et al. Safety culture and workforce well-being associations with positive leadership walkrounds. Jt Comm J Qual Patient Saf. 2021;47(7):403–11. |
| S115 | Smith 2013 | Smith R, Ooms A, Greenwood N. Supporting people with young onset dementia and their families: An evaluation of a training course for care workers. Nurs Educ Pract. 2017;27:7–12. |
| S116 | Smith 2017 | Smith S, Stevens SC. Aged care system workforce: training quality in NSW. Australas J Ageing. 2013;32(4):233–5. |
| S117 | Smith-Carrier 2015 | Smith-Carrier T, Pham TN, Akhtar S, Nowaczynski M, Seddon G, Sinha S. “A more rounded full care model”: interprofessional team members’ perceptions of home-based primary care in Ontario, Canada. Home Health Care Serv Q. 2015;34(3/4):232–51. |
| S118 | Smyth 2015 | Smyth EEJ. Assessing the skills of home care workers in helping older people take their prescribed medications. Br J Community Nurs. 2015;20(8):400–4. |
| S119 | Sogstad 2020 | Sogstad M, Hellesø R, Skinner MS. The development of a new care service landscape in Norway. Health Serv Insights. 2020;13:1–7. |
| S120 | Squillace 2009 | Squillace MR, Remsburg RE, Harris-Kojetin LD, Bercovitz A, Rosenoff E, Han B. The national nursing assistant survey: Improving the evidence base for policy initiatives to strengthen the certified nursing assistant workforce. Gerontologist. 2009;49(2):185–97. |
| S121 | Suter 2017 | Suter E, Deutschlander S, Makwarimba E, Wilhelm A, Jackson K, Lyons SW. Workforce utilization in three continuing care facilities. Health Sociol Rev. 2014;23(1):65–76. |
| S122 | Syson 2018 | Syson G, Bond J. Integrating health and social care teams in Salford. J Integr Care. 2010;18(2):17–24. |
| S123 | Szczepura 2023 | Szczepura A, Masaki H, Wild D, Nomura T, Collinson M, Kneafsey R. Integrated long-term care 'neighbourhoods' to support older populations: Evolving strategies in Japan and England. Int J Environ Res Public Health. 2023;20(14). |
| S124 | Temkin-Greener 2020 | Temkin-Greener H, Szydlowski J, Intrator O, Olsan T, Karuza J, Cai X, et al. Perceived effectiveness of home-based primary care teams in veterans health administration.. Gerontologist. 2020;60(3):494–502. |
| S125 | Tsui 2022 | Tsui EK, Franzosa E, Reckrey JM, LaMonica M, Cimarolli VR, Boerner K. Interventions to reduce the impact of client death on home care aides: Employers' perspectives. J Appl Gerontol. 2022;41(2):332–40. |
| S126 | Tullar 2016 | Tullar JM, Amick III BC, Brewer S, Diamond PM, Kelder SH, Mikhail O. Improve employee engagement to retain your workforce. Health Care Manage Rev. 2016;41(4):316–24. |
| S127 | Tveito 2009 | Tveito TH, Eriksen HR. Integrated health programme: A workplace randomized controlled trial. J Adv Nurs. 2009;65(1):110–9. |
| S128 | Tyler 2022 | Tyler DA, Squillace MR, Porter KA, Hunter M, Haltermann W. Covid-19 exacerbated long-standing challenges for the home care workforce. J Aging Soc Policy. 2022;(8914669, c57):1–19. |
| S129 | Udesen 2021 | Udesen SEJ, Lassen AT, Andersen N, Østervang C, Nielsen DS. Healthcare professionals’ experiences with highly qualified nurses working in acute care teams in primary healthcare settings. Scand J Prim Health. 2021;39(2):194–203. |
| S130 | van der Borg 2017 | van der Borg WE, Verdonk P, Dauwerse L, Abma TA. Work-related change in residential elderly care: Trust, space and connectedness. Hum Relat. 2017;70(7):805–35. |
| S131 | van der Kooij 2013 | van der Kooij C, Dröes R, de Lange J, Ettema T, Cools H, van Tilburg W. The implementation of integrated emotion-oriented care: Did it actually change the attitude, skills and time spent of trained caregivers? Dementia. 2013;12(5):536–50. |
| S132 | van Haeften-van Dijk 2017 | van Haeften-van DijkDijk AM, Hattink BJJ, Meiland FJM, Bakker TJEM, Dröes RM. Is socially integrated community day care for people with dementia associated with higher user satisfaction and a higher job satisfaction of staff compared to nursing home-based day care? Aging Ment Health. 2017;21(6):624–33. |
| S133 | van Weert 2005 | van Weert ia CM, van Dulmen AM, Spreeuwenberg PMM, Ribbe MW, Bensing JM. Effects of snoezelen, integrated in 24h dementia care, on nurse-patient communication during morning care. Patient Educ Couns. 2005;58(3):312–26. |
| S134 | VerValin 2018 | VerValin J, Wu G, Zhang Y. Impact of increasing wages for home health care workers in New York State [Internet]. Ithaca NY: Cornell University; 2018 May [cited 2024 Jul 9] p. 46. Available from: <https://www.nysenate.gov/sites/default/files/cipa_capstone_final_report_office_of_aging2613.pdf> |
| S135 | Warmoth 2022 | Warmoth K., Lynch J., Darlington N., Bunn F., Goodman C. Using video consultation technology between care homes and health and social care professionals: a scoping review and interview study during COVID-19 pandemic. Age Ageing. 2022;51(2):afab279. |
| S136 | Warmoth 2023 | Warmoth K, Bennett C, Lynch J, Goodman C. Using online consultations to facilitate health and social care delivery during covid-19: An interview study of care home staff.. J Long Term Care. 2023;100–8. |
| S137 | Wilberforce 2023 | Wilberforce M, Dunn A, Tiffin PA. Recruiting a person-centered direct care workforce through situational judgement tests: A pilot study in the community support of older people in England. Innov Aging. 2023;7(9):igad112. |
| S138 | Wild 2011 | Wild D, Szczepura A, Nelson S. How social care staff working in residential homes perceive their professional status. Nurs Older People. 2011;23(7):29–35. |
| S139 | Wilkinson 2021 | Wilkinson EK, Lees A, Weekes S, Duncan G, Meads G, Tapson K. A collaborative, multi-sectoral approach to implementing a social prescribing initiative to alleviate social isolation and enhance well-being amongst older people. J Integr Care. 2021;29(1):37–47. |
| S140 | Woodward 2023 | Woodward A, Ruston A. Empowerment of care home staff through effective collaboration with healthcare. J Interprof Care. 2023;37(1):109–17. |
| S141 | Woolrych 2013 | Woolrych R, Sixsmith J. Toward integrated services for dementia: A formal carer perspective. J Integr Care. 2013;21(4):208–20. |
| S142 | Wu 2021 | Wu SC, Peng MC, Hsueh JY, Chiang TL, Tu YK, Tung YC, et al. Impact of a new home care payment mechanism on growth of the home care workforce in Taiwan. Gerontologist. 2021;61(4):505–16. |
| S143 | Yan 2023 | Yan D, Temkin-Greener H, Pavan R, Yu H, Cai S. Did minimum wage policy changes impact home health workforce? Home Health Care Manag Pract. 2023;35(3):206–12. |
| S144 | Young 2023 | Young CC, Kesler S, Walker VG, Johnson A, Harrison TC. An online mindfulness-based intervention for certified nursing assistants in long-term care. J Holist Nurs. 2023;41(2):130–41. |
| S145 | Zeilig 2015 | Zeilig H, Poland F, Fox C, Killick J. The arts in dementia care education: A developmental study. J  Public Ment Health. 2015;14(1):18–23. |
| Search Update: Sources added November 2024 | | |
| S146 | Crevacore 2024 | Crevacore C, Coventry L, Duffield C, Jacob E. Factors impacting nursing assistants to accept a delegation in the acute care settings: A mixed method study. J Clin Nurs. 2024 Jun;33(6):2153–64. |
| S147 | Kelly 2024 | Kelly C, Dansereau L, Sebring JCH, Lee Y, Williams A. Wages or legitimacy? A qualitative analysis of home care worker perspectives in choosing work settings. Inquiry. 2024;61. |
| S148 | McKay 2024 | McKay S, Konan M, Tedesco S, Turriff T, Michener M, King EC. Optimizing weekend schedules in home health care: the essential care on weekends for personal support quality improvement project. Home Health Care Manag Pract. 2024 May 1;36(2):81–7. |
| S149 | Roth 2024 | Roth SE, Marsi K, Kenton N, Cohen-Cline H. Supporting stabilization: a qualitative evaluation of a pilot program to integrate personal caregiving services into housing settings. Inquiry. 2024 Jan;61. |
| S150 | SfC 2024 | Fenton W, Polzin G, Fleming N, Fozzard T, Price R, Davison S, et al. Skills for Care, The state of the adult social care sector and workforce in England, 2024 [Internet]. Leeds UK: Skills for Care; 2024 [cited 2024 Nov 21] p. 170. Available from: <https://www.skillsforcare.org.uk/Adult-Social-Care-Workforce-Data/Workforce-intelligence/documents/State-of-the-adult-social-care-sector/The-state-of-the-adult-social-care-sector-and-workforce-in-England-2024.pdf> |
| S151 | Varese 2024 | Varese F, Allsopp K, Carter LA, Shields G, Hind D, Davies L, et al. The Resilience Hub approach for addressing mental health of health and social care workers during the COVID-19 pandemic: a mixed-methods evaluation. Health & Social Care Deliv Research. 2024;12(29):1–164. |

# Source by thematic areas of interest based on the Working Lifespan framework

As can be seen in the table below, most of the sources informed multiple areas of interest (e.g., S108 Rödlach 2009 touched on 11 themes, S87 Murphy 2022 and S46 Fischer 2020 on 10 themes, and so on).

| **WORKING LIFESPAN STAGE** | | | **Country** | **Entry** | | | | | | | | | | | | | | **Supporting/Working** | | | | | | | | | | | | | | **Exit** | | | | **X-Cutting** | | | |
| --- | --- | --- | --- | --- | --- | --- | --- | --- | --- | --- | --- | --- | --- | --- | --- | --- | --- | --- | --- | --- | --- | --- | --- | --- | --- | --- | --- | --- | --- | --- | --- | --- | --- | --- | --- | --- | --- | --- | --- |
| **Source ID** | | **Source** |  | Data & evidence | Needs identification | | Governance | | Funding | | Pre-service Education | | Recruitment | | Professional regulation | | Org. leadership | | Working conditions | | Scope of practice | | Support systems | | Compensation - benefits | | Life-long learning | | Career advancement | | Community integration | | Retirement / Retention | | Career choice/ pathways | | Occ. Segregation, equity | | Public & Private |
| S1 | | Aldeghi 2013 | France |  | |  | |  | |  | |  | |  | | X | |  | |  | |  |  |  | |  | | X | |  | | X | |  | |  | |  | |
| S2 | | Arain 2017 | Canada |  | |  | |  | |  | | X | |  | |  | |  | |  | | X |  |  | |  | |  | |  | |  | |  | |  | |  | |
| S3 | | Ashwood 2016 | UK |  | |  | |  | |  | |  | |  | |  | | X | |  | | X | X |  | | X | |  | |  | | X | |  | |  | |  | |
| S4 | | Australia 2021 | Australia | X | |  | | X | |  | |  | |  | |  | |  | |  | | X | X | X | | X | | X | | X | |  | | X | |  | |  | |
| S5 | | Bandini 2024 | USA |  | |  | |  | |  | |  | |  | |  | |  | |  | |  |  |  | |  | |  | |  | | X | |  | | X | |  | |
| S6 | | Barnett 2018 | Australia |  | |  | |  | | X | | X | |  | |  | | X | |  | |  |  |  | | X | | X | |  | |  | |  | |  | |  | |
| S7 | | Basnight 2023 | USA |  | |  | |  | |  | | X | | X | |  | |  | |  | | X |  |  | |  | |  | |  | |  | |  | |  | |  | |
| S8 | | Baughman 2010 | USA |  | |  | |  | |  | |  | |  | |  | |  | |  | |  |  | X | |  | |  | |  | |  | |  | |  | |  | |
| S9 | | Bayly 2018 | Canada |  | |  | |  | |  | |  | |  | |  | | X | |  | |  |  |  | | X | |  | | X | |  | |  | |  | |  | |
| S10 | | Bernard 2005 | UK |  | |  | | X | |  | |  | | X | |  | |  | |  | |  |  |  | |  | |  | |  | |  | |  | |  | |  | |
| S11 | | Bjerregaard 2015 | UK |  | |  | |  | |  | |  | |  | |  | | X | |  | |  |  | X | | X | |  | |  | |  | |  | |  | |  | |
| S12 | | Boscart 2018 | Canada |  | |  | |  | |  | |  | |  | |  | |  | |  | |  |  |  | | X | |  | |  | |  | |  | |  | |  | |
| S13 | | Boscart 2019 | Canada |  | |  | |  | |  | |  | |  | |  | |  | |  | |  |  |  | | X | |  | |  | |  | |  | |  | |  | |
| S14 | | Boult 2011 | USA |  | |  | |  | | X | |  | |  | | X | | X | |  | |  |  |  | |  | |  | |  | |  | |  | |  | |  | |
| S15 | | Boumans 2008 | Netherlands |  | |  | |  | |  | |  | |  | |  | | X | | X | | X |  |  | |  | |  | |  | |  | |  | |  | |  | |
| S16 | | Brown 2016 | USA |  | |  | |  | |  | |  | |  | |  | |  | | X | |  |  |  | |  | |  | |  | | X | |  | |  | |  | |
| S17 | | Bunn 2020 | UK |  | |  | |  | |  | |  | |  | |  | |  | |  | | X |  |  | |  | | X | |  | |  | |  | |  | |  | |
| S18 | | Burgdorf 2020 | USA |  | |  | |  | | X | |  | | X | |  | |  | |  | |  |  |  | |  | |  | |  | |  | |  | |  | |  | |
| S19 | | Chapman 2023 | USA |  | |  | |  | |  | |  | | X | |  | | X | |  | |  | X |  | | X | |  | |  | | X | |  | |  | |  | |
| S20 | | Charlesworth 2024 | Multi |  | |  | |  | |  | |  | |  | |  | |  | |  | |  |  | X | |  | |  | |  | |  | |  | | X | | X | |
| S21 | | Chen 2014 | Multi |  | |  | |  | |  | | X | | X | |  | |  | | X | |  |  | X | | X | | X | |  | | X | |  | | X | | X | |
| S22 | | Chen 2020 | Taiwan |  | |  | | X | | X | |  | | X | |  | |  | |  | |  |  | X | |  | |  | |  | |  | |  | | X | |  | |
| S23 | | Chester 2014 | UK |  | |  | | X | |  | |  | | X | |  | |  | |  | |  |  |  | |  | |  | |  | | X | |  | |  | |  | |
| S24 | | Choy 2016 | Australia |  | |  | |  | |  | |  | |  | |  | |  | |  | |  |  |  | | X | | X | |  | |  | |  | |  | |  | |
| S25 | | Christensen 2017 | Multi |  | |  | | X | |  | |  | | X | |  | |  | |  | |  |  |  | |  | |  | |  | |  | |  | | X | |  | |
| S26 | | Clapper 2023 | Netherlands |  | |  | | X | |  | |  | |  | |  | |  | |  | | X |  |  | |  | |  | |  | |  | |  | |  | |  | |
| S27 | | Clarke 2019 | UK |  | |  | |  | |  | |  | |  | |  | |  | |  | | X |  |  | | X | |  | |  | |  | |  | |  | |  | |
| S28 | | Coogle 2007 | USA |  | |  | |  | |  | |  | |  | |  | |  | |  | |  |  |  | | X | |  | |  | | X | |  | |  | |  | |
| S29 | | Cook 2017 | UK |  | |  | |  | |  | |  | |  | |  | |  | |  | |  | X |  | |  | |  | |  | |  | |  | |  | | X | |
| S30 | | Craswell 2020 | Australia |  | |  | |  | | X | |  | |  | |  | |  | |  | | X |  |  | |  | | X | |  | |  | |  | |  | |  | |
| S31 | | DeGraves 2024 | Canada |  | |  | |  | |  | |  | |  | |  | |  | |  | |  | X |  | |  | |  | |  | |  | |  | |  | |  | |
| S32 | | Denton 2015 | Canada |  | |  | |  | |  | |  | |  | |  | |  | |  | | X |  |  | |  | |  | |  | |  | |  | |  | |  | |
| S33 | | Deshong 2010 | Australia |  | |  | |  | |  | | X | |  | |  | |  | |  | | X |  | X | | X | | X | |  | |  | |  | |  | |  | |
| S34 | | Devine 2006 | UK |  | |  | |  | |  | |  | |  | |  | | X | |  | |  |  |  | |  | |  | | X | |  | |  | |  | |  | |
| S35 | | Dill 2010 | USA |  | |  | |  | |  | |  | |  | |  | | X | |  | |  |  | X | | X | |  | |  | | X | |  | |  | | X | |
| S36 | | Dill 2014 | USA |  | |  | |  | |  | |  | |  | |  | |  | |  | |  |  | X | |  | | X | |  | |  | |  | |  | |  | |
| S37 | | Dill 2022 | USA |  | |  | |  | |  | |  | | X | |  | |  | |  | |  |  | X | |  | | X | |  | |  | | X | |  | |  | |
| S38 | | Dix 2023 | Australia |  | |  | | X | |  | |  | |  | |  | |  | | X | | X |  |  | | X | |  | |  | |  | |  | |  | |  | |
| S39 | | Douglas 2023 | USA |  | |  | |  | | X | |  | |  | |  | | X | | X | | X |  |  | | X | |  | |  | |  | |  | |  | |  | |
| S40 | | Dreher 2019 | USA |  | |  | |  | |  | |  | |  | |  | |  | |  | |  | X |  | | X | |  | |  | | X | |  | |  | |  | |
| S41 | | Dryden 2009 | UK |  | |  | |  | |  | |  | |  | |  | |  | |  | | X |  |  | | X | |  | |  | |  | |  | |  | |  | |
| S42 | | Edes 2014 | USA |  | |  | |  | | X | |  | |  | |  | |  | |  | |  |  |  | |  | |  | |  | |  | |  | |  | |  | |
| S43 | | Elbourne 2015 | UK |  | | X | | X | |  | |  | |  | |  | | X | | X | |  |  |  | | X | |  | |  | |  | |  | |  | | X | |
| S44 | | Feldman 2019 | USA |  | |  | |  | |  | | X | |  | |  | |  | | X | |  |  | X | | X | | X | |  | | X | |  | |  | |  | |
| S45 | | Finnema 2005 | Netherlands |  | |  | |  | |  | |  | |  | |  | |  | |  | |  | X |  | |  | |  | |  | |  | |  | |  | |  | |
| S46 | | Fischer 2020 | USA | X | |  | | X | | X | | X | | X | |  | |  | | X | |  | X | X | | X | | X | |  | |  | |  | |  | |  | |
| S47 | | Florek 2022 | Multi |  | |  | |  | |  | |  | |  | | X | |  | |  | |  |  |  | |  | |  | |  | |  | |  | |  | |  | |
| S48 | | Fong 2022 | USA |  | |  | |  | |  | |  | |  | |  | |  | |  | |  |  |  | | X | |  | |  | |  | |  | |  | |  | |
| S49 | | Franzosa 2023 | USA |  | |  | |  | |  | |  | |  | |  | |  | |  | |  | X |  | |  | |  | |  | |  | |  | |  | |  | |
| S50 | | Gaber 2020 | Canada |  | |  | |  | |  | | X | |  | |  | | X | |  | | X | X |  | |  | | X | | X | |  | |  | |  | |  | |
| S51 | | Gleason 2024 | USA |  | |  | |  | |  | |  | |  | |  | |  | | X | | X | X |  | |  | | X | |  | |  | |  | |  | |  | |
| S52 | | Guerrero 2020 | USA |  | |  | |  | |  | |  | |  | |  | |  | |  | |  |  |  | | X | |  | |  | |  | |  | |  | |  | |
| S53 | | Håland 2021 | Norway |  | | X | | X | |  | |  | |  | |  | | X | |  | | X | X |  | |  | |  | | X | |  | |  | |  | |  | |
| S54 | | Hald 2021 | Denmark |  | |  | |  | |  | |  | |  | |  | | X | |  | |  |  |  | |  | |  | |  | |  | |  | |  | |  | |
| S55 | | Hamer 2018 | UK |  | |  | |  | |  | |  | |  | |  | |  | |  | |  | X |  | | X | |  | |  | |  | |  | |  | |  | |
| S56 | | Hanlon 2007 | Canada |  | |  | | X | | X | |  | |  | |  | |  | |  | |  |  |  | |  | |  | |  | |  | |  | |  | |  | |
| S57 | | Hanssen 2017 | Norway |  | |  | |  | |  | |  | |  | |  | | X | |  | |  |  |  | | X | |  | |  | |  | |  | |  | |  | |
| S58 | | Harlock 2020 | UK |  | |  | | X | | X | |  | |  | |  | | X | |  | |  |  |  | |  | |  | |  | |  | |  | |  | |  | |
| S59 | | Hjelle 2016 | Norway |  | |  | |  | |  | |  | |  | |  | | X | | X | | X | X |  | |  | |  | |  | |  | |  | |  | |  | |
| S60 | | Hjelm 2000 | Sweden |  | | X | |  | |  | |  | |  | |  | |  | |  | | X | X |  | | X | |  | |  | |  | |  | |  | |  | |
| S61 | | Hollinger-Smith 2001 | USA |  | |  | |  | |  | |  | |  | |  | | X | |  | |  |  |  | |  | |  | |  | |  | |  | |  | |  | |
| S62 | | Huang 2020 | USA |  | |  | |  | |  | |  | |  | |  | |  | |  | |  |  | X | |  | |  | |  | | X | |  | |  | | X | |
| S63 | | Hult 2023 | Finland |  | |  | |  | |  | |  | |  | |  | |  | | X | |  |  |  | |  | |  | |  | |  | |  | | X | |  | |
| S64 | | Kemper 2008 | USA |  | |  | | X | |  | |  | | X | |  | | X | |  | |  |  |  | |  | |  | |  | |  | |  | |  | |  | |
| S65 | | Khavjou 2024 | USA |  | |  | | X | |  | |  | |  | |  | |  | |  | |  |  | X | |  | |  | |  | |  | |  | |  | |  | |
| S66 | | Kim 2019 | USA |  | |  | | X | |  | |  | |  | |  | |  | |  | |  |  | X | |  | |  | |  | |  | |  | |  | | X | |
| S67 | | Kornas 2021 | Canada |  | |  | |  | |  | |  | |  | |  | | X | | X | |  |  |  | | X | |  | |  | |  | |  | |  | |  | |
| S68 | | Kroezen 2018 | Multi |  | |  | |  | |  | | X | |  | |  | |  | |  | |  |  |  | | X | | X | |  | |  | |  | |  | |  | |
| S69 | | Kubo 2014 | Japan |  | |  | | X | | X | | X | | X | |  | |  | |  | |  |  |  | |  | |  | |  | |  | |  | |  | | X | |
| S70 | | Kulnik 2017 | UK |  | |  | |  | |  | |  | |  | |  | | X | |  | |  |  |  | | X | |  | |  | |  | |  | |  | |  | |
| S71 | | Lacher 2015 | Switzerland |  | |  | |  | |  | |  | |  | | X | |  | |  | |  |  | X | |  | |  | |  | |  | | X | |  | |  | |
| S72 | | Larsson 2014 | Sweden |  | |  | |  | |  | |  | |  | |  | | X | | X | |  | X |  | |  | |  | |  | |  | |  | |  | |  | |
| S73 | | Lawlis 2016 | Australia |  | |  | |  | |  | | X | |  | |  | |  | |  | |  |  |  | | X | |  | |  | |  | |  | |  | |  | |
| S74 | | Lea 2023 | Australia |  | |  | |  | |  | |  | | X | | X | |  | | X | | X |  | X | | X | | X | |  | |  | |  | |  | |  | |
| S75 | | Lee 2015 | Australia |  | |  | |  | |  | |  | |  | |  | |  | |  | | X |  |  | |  | |  | |  | |  | |  | |  | |  | |
| S76 | | Lokmic-Tomkins 2021 | Australia |  | |  | |  | |  | |  | |  | |  | |  | |  | | X |  |  | |  | | X | |  | |  | | X | |  | |  | |
| S77 | | LTSS 2018 | Multi |  | |  | |  | |  | | X | | X | |  | |  | |  | |  |  |  | |  | |  | |  | |  | |  | | X | |  | |
| S78 | | Luz 2015 | USA |  | |  | |  | |  | |  | |  | |  | |  | |  | |  |  |  | | X | |  | |  | |  | |  | | X | |  | |
| S79 | | Manheim 2021 | USA |  | |  | |  | |  | |  | |  | |  | |  | |  | |  | X |  | |  | |  | | X | |  | |  | |  | |  | |
| S80 | | McDaniel 2011 | USA |  | |  | |  | |  | |  | |  | |  | | X | | X | |  |  |  | |  | |  | |  | | X | |  | |  | |  | |
| S81 | | Merkel 2019 | Multi |  | |  | |  | |  | |  | | X | |  | |  | | X | |  |  |  | | X | | X | |  | | X | |  | |  | |  | |
| S82 | | Meyer 2018 | Australia |  | |  | |  | |  | |  | |  | |  | |  | |  | |  |  |  | | X | |  | |  | |  | |  | |  | |  | |
| S83 | | Monro 2021 | Australia |  | |  | |  | | X | | X | |  | |  | |  | | X | | X |  |  | | X | |  | |  | |  | |  | |  | |  | |
| S84 | | Morgan 2008 | USA |  | |  | |  | |  | |  | |  | |  | | X | | X | |  |  | X | | X | |  | |  | | X | |  | |  | |  | |
| S85 | | Morgan 2018 | USA | X | |  | | X | |  | | X | | X | | X | |  | |  | |  |  | X | | X | | X | |  | |  | |  | |  | |  | |
| S86 | | Mun 2023 | South Korea |  | |  | | X | | X | |  | |  | |  | | X | |  | |  | X |  | | X | |  | |  | |  | |  | |  | |  | |
| S87 | | Murphy 2022 | Multi |  | |  | | X | |  | | X | | X | | X | |  | | X | |  |  | X | | X | | X | |  | |  | |  | | X | |  | |
| S88 | | Naccarella 2018 | Australia |  | |  | |  | |  | |  | |  | |  | |  | | X | |  |  |  | |  | |  | |  | |  | |  | |  | |  | |
| S89 | | Nadav 2021 | Finland |  | |  | |  | |  | |  | |  | |  | |  | |  | |  | X |  | |  | |  | |  | |  | |  | |  | |  | |
| S90 | | Nandram 2014 | Netherlands |  | |  | | X | | X | |  | |  | |  | | X | | X | | X | X |  | |  | |  | |  | | X | |  | |  | |  | |
| S91 | | Navarra 2023 | Multi | X | |  | | X | |  | |  | |  | |  | |  | | X | |  | X | X | |  | |  | |  | |  | |  | | X | |  | |
| S92 | | Norman 2018 | USA |  | |  | |  | | X | | X | | X | |  | |  | |  | |  | X |  | |  | |  | |  | | X | |  | |  | |  | |
| S93 | | Obayashi 2020 | Japan |  | |  | |  | |  | |  | |  | |  | |  | |  | | X | X |  | |  | |  | |  | |  | |  | |  | |  | |
| S94 | | Ochieng 2022 | UK |  | |  | |  | |  | |  | |  | |  | |  | |  | | X | X |  | | X | |  | |  | |  | |  | |  | |  | |
| S95 | | Ochylski 2017 | USA |  | |  | |  | |  | | X | |  | |  | |  | |  | |  |  |  | |  | |  | |  | |  | |  | |  | |  | |
| S96 | | Ornstein 2011 | USA |  | |  | |  | | X | |  | |  | |  | | X | |  | |  | X |  | |  | |  | |  | |  | |  | |  | |  | |
| S97 | | Øvretveit 2010 | Sweden |  | |  | | X | | X | |  | |  | |  | | X | |  | |  |  |  | |  | |  | |  | |  | |  | |  | |  | |
| S98 | | Pagaiya 2021 | Thailand |  | |  | |  | |  | | X | | X | |  | |  | | X | | X |  | X | |  | |  | |  | |  | |  | |  | |  | |
| S99 | | Parveen 2021 | UK |  | |  | |  | |  | | X | |  | |  | | X | | X | |  |  |  | | X | |  | |  | |  | |  | |  | |  | |
| S100 | | Paulus 2005 | Netherlands |  | |  | |  | |  | |  | |  | |  | |  | |  | |  |  |  | |  | |  | | X | |  | |  | |  | |  | |
| S101 | | Pointu 2005 | UK |  | |  | |  | |  | |  | |  | |  | |  | |  | |  |  |  | | X | |  | |  | |  | |  | |  | |  | |
| S102 | | Poulain 2023 | India |  | |  | |  | |  | | X | | X | |  | |  | |  | |  |  |  | |  | |  | |  | |  | |  | | X | |  | |
| S103 | | Reymond 2005 | Australia |  | |  | |  | |  | |  | |  | |  | |  | |  | |  |  |  | | X | |  | | X | |  | |  | |  | |  | |
| S104 | | Robben 2012 | Netherlands |  | |  | |  | |  | |  | |  | |  | |  | |  | |  | X |  | |  | |  | |  | |  | |  | |  | |  | |
| S105 | | Robertson 2023 | UK |  | |  | |  | |  | | X | |  | |  | |  | |  | | X |  |  | |  | |  | |  | |  | |  | |  | |  | |
| S106 | | Robyn 2015 | Cameroon |  | |  | |  | |  | |  | | X | |  | |  | | X | |  |  |  | |  | |  | |  | |  | |  | |  | |  | |
| S107 | | Rodgers 2017 | New Zealand |  | |  | |  | |  | |  | |  | |  | | X | |  | | X | X |  | | X | |  | |  | |  | |  | |  | |  | |
| S108 | | Rödlach 2009 | Zimbabwe |  | | X | | X | | X | | X | | X | |  | | X | | X | |  | X | X | |  | |  | | X | |  | |  | | X | |  | |
| S109 | | Røsstad 2017 | Norway |  | |  | |  | |  | |  | |  | |  | |  | |  | |  | X |  | |  | |  | |  | |  | |  | |  | |  | |
| S110 | | Russell 2022 | USA |  | |  | | X | | X | |  | | X | |  | | X | | X | |  |  | X | | X | | X | |  | | X | |  | |  | |  | |
| S111 | | Sandoz 2019 | UK |  | |  | |  | |  | |  | |  | |  | |  | |  | | X |  |  | | X | |  | |  | |  | |  | |  | |  | |
| S112 | | Savassi 2021 | Brazil |  | |  | |  | |  | |  | | X | |  | |  | |  | |  |  |  | | X | |  | |  | |  | |  | |  | |  | |
| S113 | | Schoville 2020 | USA |  | |  | |  | |  | |  | |  | |  | | X | |  | | X |  |  | |  | |  | |  | |  | |  | |  | |  | |
| S114 | | Sexton 2021 | USA |  | |  | |  | |  | |  | |  | |  | | X | | X | |  |  |  | |  | |  | |  | |  | |  | |  | |  | |
| S115 | | Smith 2013 | Australia |  | |  | |  | |  | | X | |  | |  | | X | |  | |  |  |  | |  | |  | |  | |  | |  | |  | |  | |
| S116 | | Smith 2017 | UK |  | |  | |  | |  | |  | |  | |  | | X | |  | |  |  |  | | X | |  | |  | | X | |  | |  | |  | |
| S117 | | Smith-Carrier 2015 | Canada |  | |  | |  | | X | |  | |  | |  | | X | | X | | X | X |  | | X | |  | | X | |  | |  | |  | |  | |
| S118 | | Smyth 2015 | UK |  | |  | |  | |  | | X | |  | |  | |  | |  | | X |  |  | | X | |  | |  | |  | |  | |  | |  | |
| S119 | | Sogstad 2020 | Norway |  | | X | | X | | X | | X | |  | |  | |  | |  | | X |  |  | |  | |  | |  | |  | |  | |  | |  | |
| S120 | | Squillace 2009 | USA | X | |  | |  | |  | |  | |  | |  | |  | |  | |  |  |  | |  | |  | |  | |  | |  | |  | |  | |
| S121 | | Suter 2017 | Canada |  | |  | | X | |  | |  | |  | |  | |  | |  | | X |  |  | |  | |  | |  | |  | |  | |  | | X | |
| S122 | | Syson 2018 | UK |  | |  | | X | |  | |  | |  | |  | | X | |  | | X | X |  | | X | |  | |  | |  | |  | |  | |  | |
| S123 | | Szczepura 2023 | Multi |  | |  | | X | |  | |  | |  | |  | |  | |  | |  |  |  | |  | |  | |  | |  | |  | |  | |  | |
| S124 | | Temkin-Greener 2020 | USA |  | |  | |  | |  | |  | |  | |  | | X | | X | | X |  |  | |  | |  | |  | |  | |  | |  | |  | |
| S125 | | Tsui 2022 | USA |  | |  | |  | |  | |  | |  | |  | | X | | X | |  | X | X | |  | |  | |  | |  | |  | |  | | X | |
| S126 | | Tullar 2016 | USA |  | |  | |  | |  | |  | |  | |  | |  | | X | | X |  | X | |  | |  | |  | | X | | X | |  | |  | |
| S127 | | Tveito 2009 | Norway |  | |  | |  | |  | |  | |  | |  | |  | | X | |  | X |  | |  | |  | |  | |  | |  | |  | |  | |
| S128 | | Tyler 2022 | USA |  | |  | | X | | X | | X | | X | |  | | X | | X | |  | X |  | |  | |  | |  | | X | |  | |  | |  | |
| S129 | | Udesen 2021 | Denmark |  | |  | | X | |  | |  | |  | |  | |  | | X | | X | X |  | |  | |  | | X | |  | |  | |  | |  | |
| S130 | | van der Borg 2017 | Netherlands |  | |  | |  | |  | |  | | X | |  | | X | | X | |  | X |  | |  | |  | |  | |  | |  | |  | |  | |
| S131 | | van der Kooij 2013 | Netherlands |  | |  | |  | |  | |  | |  | |  | | X | | X | |  |  |  | | X | |  | |  | |  | |  | |  | |  | |
| S132 | | van Haeften-van Dijk 2017 | Netherlands |  | |  | |  | |  | |  | |  | |  | |  | | X | |  |  |  | | X | |  | |  | |  | |  | |  | |  | |
| S133 | | van Weert 2005 | Netherlands |  | |  | |  | |  | |  | |  | |  | |  | | X | | X | X |  | | X | |  | |  | |  | |  | |  | |  | |
| S134 | | VerValin 2018 | USA | X | |  | |  | | X | |  | |  | |  | |  | |  | |  |  | X | |  | |  | |  | |  | |  | |  | |  | |
| S135 | | Warmoth 2022 | UK |  | |  | |  | |  | |  | |  | |  | |  | |  | |  | X |  | |  | |  | |  | |  | |  | |  | |  | |
| S136 | | Warmoth 2023 | UK |  | |  | |  | |  | |  | |  | |  | |  | | X | | X | X |  | | X | |  | |  | |  | |  | |  | |  | |
| S137 | | Wilberforce 2023 | UK |  | |  | |  | |  | |  | | X | |  | |  | |  | |  |  |  | |  | |  | |  | |  | |  | |  | |  | |
| S138 | | Wild 2011 | UK |  | |  | |  | |  | |  | |  | | X | |  | |  | |  |  |  | |  | |  | |  | |  | |  | |  | |  | |
| S139 | | Wilkinson 2021 | UK | X | |  | | X | | X | |  | |  | |  | | X | |  | |  | X |  | |  | |  | |  | |  | |  | |  | |  | |
| S140 | | Woodward 2023 | UK |  | |  | |  | |  | |  | |  | |  | | X | |  | |  | X |  | | X | |  | |  | |  | |  | | X | |  | |
| S141 | | Woolrych 2013 | UK |  | |  | |  | |  | |  | |  | |  | |  | | X | |  | X | X | | X | |  | |  | |  | |  | |  | |  | |
| S142 | | Wu 2021 | Taiwan |  | |  | |  | |  | |  | | X | |  | |  | | X | |  |  | X | |  | | X | |  | | X | | X | | X | |  | |
| S143 | | Yan 2023 | USA |  | |  | | X | |  | |  | | X | |  | |  | |  | |  |  | X | |  | |  | |  | | X | |  | |  | |  | |
| S144 | | Young 2023 | USA |  | |  | |  | |  | |  | |  | |  | |  | |  | |  | X |  | |  | |  | |  | |  | |  | |  | |  | |
| S145 | | Zeilig 2015 | UK |  | |  | |  | |  | |  | |  | |  | |  | |  | |  |  |  | | X | |  | |  | |  | |  | |  | |  | |
|  | Search update: Sources added November 2024 | | | | | | | | | | | | | | | | | | | | | | | | | | | | | | | | | | | | | | |
| S146 | | Crevacore 2024 | Australia |  | |  | |  | |  | |  | |  | |  | |  | | X | | X | X |  | |  | |  | |  | |  | |  | |  | |  | |
| S147 | | Kelly 2024 | Canada |  | |  | |  | |  | |  | |  | |  | |  | | X | |  |  | X | |  | |  | |  | |  | |  | | X | |  | |
| S148 | | McKay 2024 | Canada |  | |  | |  | |  | |  | |  | |  | |  | | X | |  |  |  | |  | |  | |  | |  | |  | |  | |  | |
| S149 | | Roth 2024 | USA |  | |  | |  | |  | |  | |  | |  | |  | |  | | X |  | X | |  | |  | |  | | X | |  | |  | |  | |
| S150 | | SfC 2024 | UK | X | |  | |  | | X | | X | | X | |  | |  | | X | |  |  | X | |  | | X | |  | | X | |  | | X | | X | |
| S151 | | Varese 2024 | UK |  | | X | |  | | X | |  | |  | |  | |  | |  | |  | X |  | |  | |  | |  | |  | |  | | X | |  | |
